# Metagenomic assessment of gut microbial communities and risk of severe COVID-19

**DOI:** 10.21203/rs.3.rs-1717624/v1

**Published:** 2022-06-07

**Authors:** Peggy Lai, Long Nguyen, Daniel Okin, David Drew, Vincent Battista, Sirus Jesudasen, Thomas Kuntz, Amrisha Bhosle, Kelsey Thompson, Trenton Reinicke, Chun-Han Lo, Jacqueline Woo, Alexander Caraballo, Lorenzo Berra, Jacob Vieira, Ching-Ying Huang, Upasana Das Adhikari, Minsik Kim, Hui-Yu Sui, Marina Magicheva-Gupta, Lauren McIver, Marcia Goldberg, Douglas Kwon, Curtis Huttenhower, Andrew Chan

**Affiliations:** Massachusetts General Hospital, Harvard Medical School; Massachusetts General Hospital and Harvard Medical School; Massachusetts General Hospital; Clinical and Translational Epidemiology Unit, Massachusetts General Hospital; Massachusetts General Hospital; Massachusetts General Hospital; Harvard T.H. Chan School of Public Health; Harvard TH Chan School of Public Health; Harvard T.H. Chan School of Public Health; Massachusetts General Hospital; Massachusetts General Hospital and Harvard Medical School; Massachusetts General Hospital; Massachusetts General Hospital; Massachusetts General Hospital; Massachusetts General Hospital; Massachusetts General Hospital; Massachusetts General Hospital; Massachusetts General Hospital; Massachusetts General Hospital; Massachusetts General Hospital; Harvard T.H. Chan School of Public Health; Massachusetts General Hospital; Harvard Medical School; Harvard T.H. Chan School of Public Health; Massachusetts General Hospital

## Abstract

The gut microbiome is a critical modulator of host immunity and is linked to the immune response to respiratory viral infections. However, few studies have gone beyond describing broad compositional alterations in severe COVID-19, defined as acute respiratory or other organ failure. We profiled 127 hospitalized patients with COVID-19 (n=79 with severe COVID-19 and 48 with moderate) who collectively provided 241 stool samples from April 2020 to May 2021 to identify links between COVID-19 severity and gut microbial taxa, their biochemical pathways, and stool metabolites. 48 species were associated with severe disease after accounting for antibiotic use, age, sex, and various comorbidities. These included significant in-hospital depletions of Fusicatenibacter saccharivorans and Roseburia hominis, each previously linked to post-acute COVID syndrome or “long COVID”, suggesting these microbes may serve as early biomarkers for the eventual development of long COVID. A random forest classifier achieved excellent performance when tasked with predicting whether stool was obtained from patients with severe vs. moderate COVID-19. Dedicated network analyses demonstrated fragile microbial ecology in severe disease, characterized by fracturing of clusters and reduced negative selection. We also observed shifts in predicted stool metabolite pools, implicating perturbed bile acid metabolism in severe disease. Here, we show that the gut microbiome differentiates individuals with a more severe disease course after infection with COVID-19 and offer several tractable and biologically plausible mechanisms through which gut microbial communities may influence COVID-19 disease course. Further studies are needed to validate these observations to better leverage the gut microbiome as a potential biomarker for disease severity and as a target for therapeutic intervention.

## Introduction

Over 530 million individuals worldwide have been infected with SARS-CoV-2 and developed coronavirus disease-2019 (COVID-19), culminating in more than 6 million lives lost^1^. The gut microbiome is a critical modulator of host immunity^2^ and affects the immune response to respiratory viral infections (e.g., influenza A virus subtype H1N1, Severe Acute Respiratory Syndrome [SARS], and Middle East Respiratory Syndrome)^3–6^. Several early studies have explored the link between broad alterations in gut microbial communities and COVID-19, demonstrating the generalized enrichment of opportunistic pathogens and depletion of commensals^7–18^.

Most prior studies have largely focused on the presence, absence, or the differential abundance of specific microbes in COVID-19,^7,9–16^, and few have interrogated microbial network dynamics to identify which co-occurring or co-excluded species are foundational to maintaining microbial homeostasis. This represents a missed opportunity to identify potential bacterial targets to restore a more favorable, health-promoting gut configuration. Similarly, other studies have not considered how these shifts might influence gut metabolite pools. Finally, prior studies interested in exploring the gut microbiome in COVID-19 have largely sought to characterize the differences in healthy controls compared to infected patients rather than those with moderate compared to severe disease^7, 10–12,14,16^. Establishing a predictive biomarker of disease severity may improve early identification of at-risk patient populations that require immediate intervention or those that are more likely to benefit from effective antiviral therapies^19^.

It remains unclear what role the gut microbiome plays in regulating the severity of COVID-19 in hospitalized patients and what specific microbially-mediated mechanisms may underlie this relationship. To address these questions, we conducted a study of hospitalized patients with COVID-19 at a U.S. tertiary medical center. Using metagenomic profiling of fecal samples collected from these patients, we demonstrate significant depletions of *Fusicatenibacter saccharivorans* and *Roseburia hominis in* severe COVID-19, reductions of which have previously been linked to post-acute COVID-19 syndrome (PASC) or long COVID^18,20^. Strikingly, we observed these declines during patients’ index hospitalizations, suggesting the presence of an early microbial signal that may predict the development of a long-term complication. We further use network analysis to identify several critical taxa central to maintaining a gut microbial configuration less likely to be found in severe COVID-19 and perform complementary predicted metabolite analyses to further link these changes to alterations in bile acid pool and short-chain fatty acid (SCFA) levels, offering biologically plausible mechanisms to explain the link between gut microbial communities and COVID-19 disease severity.

## Results

### Participant characteristics and overall gut community structure

**Figure 1:**
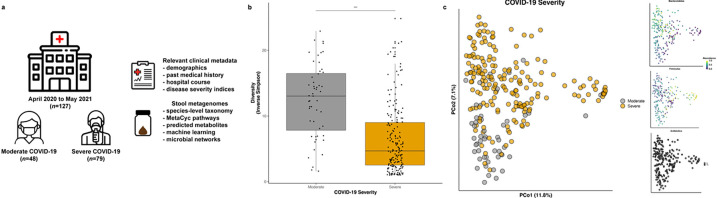
Study overview and overall community structure. **a.** Study enrollment of hospitalized patients with confirmed COVID-19 with weekly stool sampling until the time of discharge or death, whichever occurred first. **b.** Marked reduction in species richness and evenness in severe COVID-19 (inverse Simpson α-diversity metric, p-value <0.0001 from multivariable linear modeling adjusting for age, sex, prior antibiotic use, race, ethnicity, body mass index, Charlson Comorbidity Index, use of remdesivir or corticosteroids, days since admission, SARS-CoV-2 stool viral load, sequencing depth, and a participant-level random effect). Boxes represent median and interquartile range, while whiskers represent 95%ile. **c.** Community-level disturbances in severe vs. moderate COVID-19 as depicted by joint ordination and principal coordinates analysis (PCoA), not fully explained by characteristic trade-offs in Bacteroidetes/Firmicutes or prior antibiotic use.

From April 2020 to May 2021, we prospectively enrolled hospitalized patients aged ≥ 18 years with confirmed COVID-19 at the Massachusetts General Hospital to a longitudinal COVID-19 disease surveillance study. Patients were categorized as having severe COVID-19 if they required admission to the intensive care unit with acute respiratory failure (the need for oxygen supplementation ≥ 15 liters per minute (LPM), non-invasive positive pressure ventilation, or mechanical ventilation) or other organ failure (such as shock requiring vasopressor initiation)^21^. Otherwise, they were categorized as having moderate COVID-19. We enrolled 127 hospitalized COVID-19 patients. 79 (62.2%) had severe disease and 48 (37.8%) had moderate disease. Collectively, they provided 241 stool samples (Fig. 1a). No statistically significant differences were observed between severity groups based on age, sex, race, ethnicity, various comorbidities, and smoking history (Suppl. Table 1). Patients with severe COVID-19 had a higher mean body mass index (BMI) as well as Simplified Acute Physiology Score II (SAPS II)^22^ and Sequential Organ Failure Assessment (SOFA) scores^23^, each a validated clinical assessment tool to risk stratify hospitalized patients’ risk of mortality^24,25^. Severe COVID-19 patients more frequently received antibiotics, antivirals, and ICU therapies. Patients with severe COVID-19 had higher 90-day mortality compared to those with moderate disease (22.8% vs. 4.2%, *p*-value = 0.01).

Gut microbial diversity was significantly reduced in severe COVID-19 after adjusting for factors such as recent antibiotic use (Fig. 1b, p-value < 0.001). Overall gut community structure also appeared to differ based on COVID-19 disease severity (multivariable R^2^ = 2.4%, *p*-value = 0.002), a finding not fully explained by characteristic trade-offs along the Bacteroidetes/Firmicutes axes of variation or prior antibiotic usage (Fig. 1c).

### Differential abundance testing

**Figure 2:**
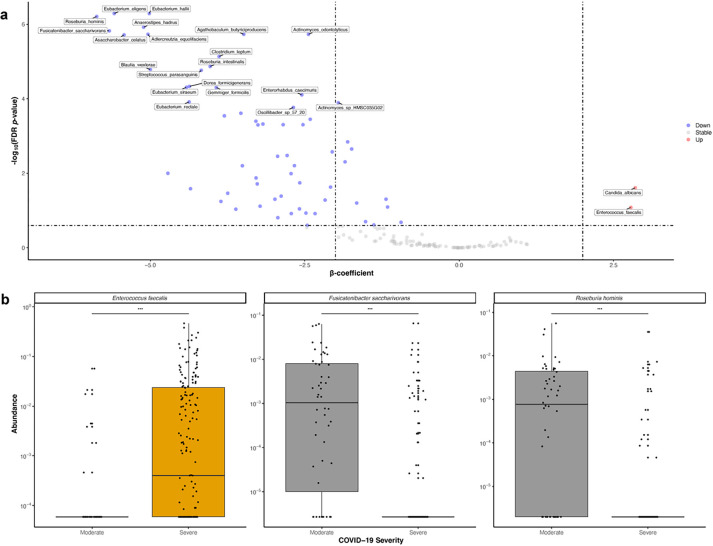
Taxonomic depletions linked to COVID-19 severity. **a.** Volcano plot of species-level expansions and depletions linked to severe vs. moderate COVID-19. Effect sizes (β-coefficients) from multivariable linear modeling plotted against FDR-corrected p-value. Full results in Suppl. Table 2. **b.** Highlighted box and scatter plots of taxa abundance by COVID-19 severity. For visualization purposes, technical/true 0s were imputed with a given taxa’s minimum non-zero value. Boxes represent median and interquartile ranges, while whiskers represent 95%ile.

Using multivariable linear mixed-effects modeling accounting for age, sex, antibiotic use, race/ethnicity, SARS-CoV-2 stool viral load and other relevant clinical metadata (Methods), we observed statistically significant differences in 48 species-level taxa between severe and moderate COVID-19 (FDR-corrected *p*-value < 0.05, Fig. 2a & Suppl. Table 2). All but two of these taxa *(Candida albicans* & *Enterococcus faecalis)* were relatively depleted in severe disease (Fig. 2a & 2b), a trend concordant with the observed decrease in species richness and evenness. We identified significant depletions of *Fusicatenibacter saccharivorans* and *Roseburia hominis* (Fig. 2b), consistent with prior work showing the relative contraction of each in patients with post-acute COVID-19 syndrome (PASC), also known as “long COVID^18,20^.” Eight taxa were positively associated with stool SARS-CoV-2 viral load, including several linked to pro-inflammatory sulfur metabolism, such as *Methanobrevibacter smithii* and *Bilophila wadsworthia*, as well as several *Alistipes* spp (Suppl. Table 2). Interestingly, an expansion of *R. hominis* was associated with increased stool viral load despite a corresponding decrease among patients with severe COVID-19, suggesting an interaction between stool SARS-CoV-2 viral load, *R. hominis,* and severe COVID-19 (Suppl. Table 2). Corresponding to community-wide depletions in microbial diversity, biochemical pathways encoded by gut bacteria were also significantly altered in severe COVID-19, including reductions in amino acid biosynthesis (e.g., glutamine synthesis), isoprenoid biosynthesis, and short-chain fatty acid production (SCFA) pathways, including glycerol degradation, acetyl-CoA fermentation, and methanogenesis from acetate (Suppl. Table 3 & Suppl. Figure 1).

### Machine learning to predict severe COVID-19

Given our findings of both community-wide and feature-level alterations linked to severe COVID-19, we next used a machine learner to predict whether metagenomic features could serve as inputs to classify samples derived from patients with severe vs. moderate COVID-19. To assess whether non-microbial metadata (i.e., participant characteristics) should be jointly considered with microbial taxa in training our classifier, we generated an entropy heatmap to quantify the unique row-wise information with respect to column-wise data (in which non-informative variables would have a value of 0). As all the covariates used in our prior linear modeling (Methods) contributed unique information to label/disease severity prediction (Suppl. Figure 2), each was included in our machine learning workflow.

**Figure 3:**
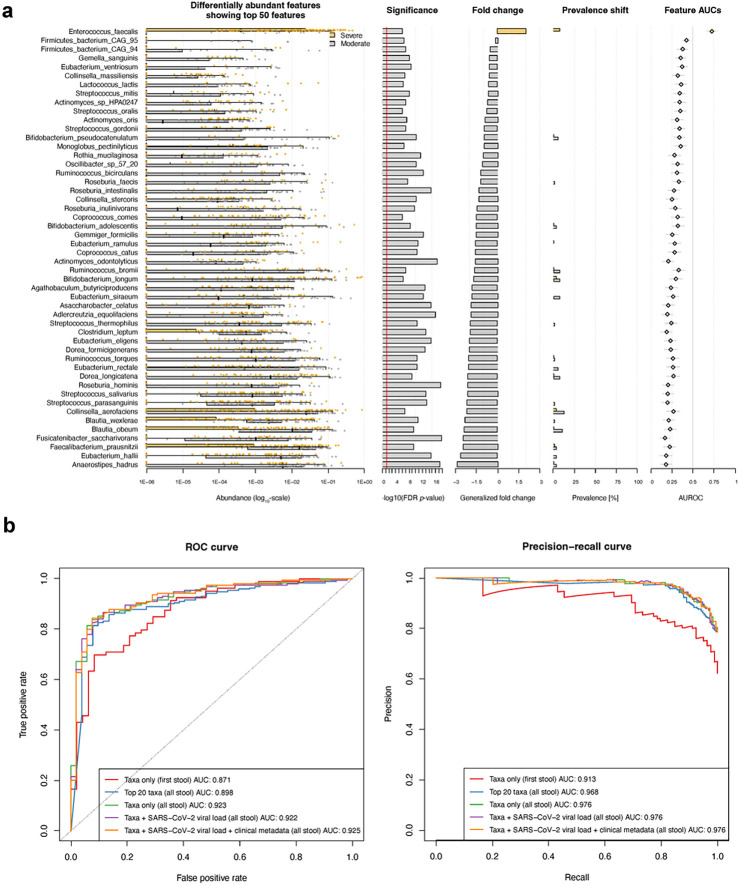
Stool-based classifier for COVID-19 disease severity. **a.** Box and scatter plots of the top 50 microbial features and their differential abundance by COVID-19 severity with barplots indicating univariate/nominal *p*-value, fold change by study group, prevalence, and taxa-level contribution to area-under-the curve for a random forest-based machine learner. **b.** Receiver operator characteristic (ROC) and precision-recall curves demonstrating excellent performance in classifying stool samples by COVID-19 severity. The removal of stool SARS-CoV-2 viral load and clinical metadata resulted in only modestly decreased task performance, as did limiting our input to only the top 20 differentially abundant microbes by disease class. A sensitivity analysis using only the first provided stool from each participant, which should minimize the possibility of overfitting data due to repeated measures and longitudinal sampling, still performed well.

Using both differentially abundant microbial features and clinical characteristics as our input (Fig. 3a), our random forest regressor achieved an area under the receiver operating characteristic (AUROC) of 0.925 when tasked with predicting whether stool was obtained from patients with severe vs. moderate COVID-19 (Fig. 3b & Fig. 3c). Our findings were only modestly attenuated when modeled without clinical metadata (AUROC 0.922) and stool SARS-CoV-2 viral load (AUROC 0.923), respectively. To robustly assess this result, we trained our model using only the top 20 differentially abundant microbial features, which only modestly degraded task performance (AUROC 0.898). Finally, though we ensured samples from the same individual were confined to a single cross-fold, to minimize the possibility of overfitting data from personalized gut microbial communities, we trained and assessed our model using only the first stool sample from each participant, which again performed with excellent accuracy (AUROC 0.871), further supporting the role of metagenomic profiling as a diagnostic biomarker for disease severity.

### Systems approaches to interrogate microbial assemblages

To explore the possible biological mechanisms underlying our observations, we next sought to characterize whether ecological networks were significantly altered based on COVID-19 severity (Methods). We hypothesized that the community-wide and feature-level alterations observed in moderate vs. severe COVID-19 would change microbial network topology. First, we evaluated global microbial network properties. The adjusted Rand Index (ARI) is a measure of similarities in clustering, quantifying the likelihood that pairs of microbial clades would be assigned to the same cluster in both networks. An ARI value of 0 indicates random clustering across comparator groups, a value of 1 indicates identical clustering, and a value of −1 indicates perfect disagreement^26,27^. When comparing moderate to severe COVID-19, the ARI was 0.199 (*p*-value < 0.001). Jaccard’s index (JI) evaluates differences among central nodes between our two severity-specific networks, where a value of 0 indicates completely different sets of central nodes and a value of 1 indicates identical central nodes^28^. While there were no statistically significant differences in overall centrality measures when comparing moderate to severe cases, there were alterations in proportion of positive edges network-wide (92.9% vs 100%, *p*-value < 0.001), indicating a loss of moderate negative correlations in severe COVID-19. For example, *C. albicans,* which was relatively more abundant in severe compared to moderate COVID-19, has 0 vs. 3 negative edges in each disease state, respectively, raising the possibility that the loss of negative selective pressure can promote the growth of certain microbial clades in severe COVID-19.

**Figure 4:**
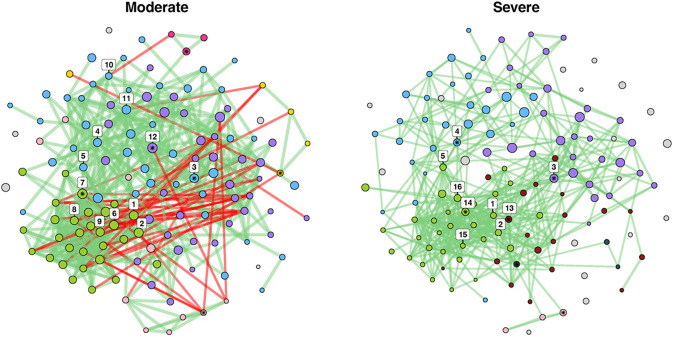
Comparative microbial assemblages in moderate vs. severe COVID-19. We assembled discrete microbial networks for moderate vs. severe disease to demonstrate significant ecological heterogeneity characterized by fractured clustering and taxa-level reassignment in severe disease. Species are represented by circles (nodes) and species-species correlations were weighted by strength of correlation (edges drawn if absolute Pearson’s ρ>0.4). Node size indicates normalized relative abundance, and node colors indicate cluster membership. Cluster colors are retained across networks if two or more taxa are shared. Edge color reflects the direction of correlation, with red edges indicating a negative, and green edges indicating a positive correlation, respectively. Hubs have been numbered, while clusters are referred to by their nominate node, or the taxa with the highest edge count in a given cluster by network.

We identified 16 taxa as network hubs, i.e., species with high putative importance given their centrality to the surrounding microbial networks (Fig. 4 & Suppl. Table 4a). Five species were identified as hubs in both moderate and severe disease *(Blautia wexlerae, Eubacterium hallii, Gordonibacter pamelaeae, Odoribacter splanchnicus,* and *Alistipes shahii),* while 11 were unique to one network or the other (Suppl. Table 4b, Suppl. Table 4c, & Suppl. Figure 3). Critically, 9 of these 16 identified hubs, including *Blautia wexlerae and Eubacterium hallii,* were shown to be differentially abundant by disease severity (Suppl. Table 2), and the relative abundance of two hubs, *Eubacterium rectale* and *Alistipes putredenis,* were associated with stool viral load. We further observed that highly-connected clusters in moderate disease become fragmented in severe COVID-19, as evidenced by an increase in singletons, a decrease in the number of hubs, and dynamic taxa-level cluster reassignment (Fig. 4). Notably, all but one of the hubs shown to be differentially abundant by disease severity belonged to the same cluster, suggesting that significant loss of these central taxa in severe disease may contribute to the observed network instability.

### Predicted stool metabolites linked to disease severity

**Figure 5:**
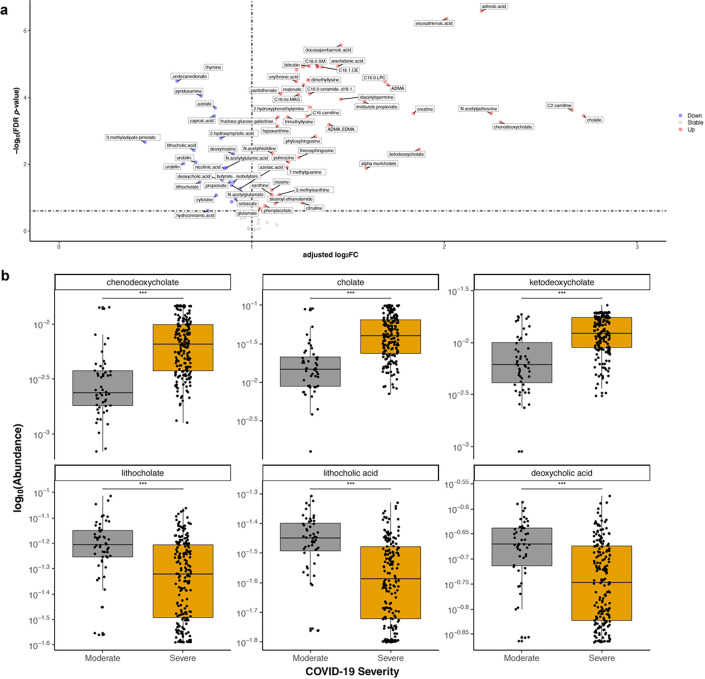
Predicted stool metabolite profiles. **a.** Volcano plot of enrichments and depletions in predicted stool metabolites linked to severe compared to moderate COVID-19. Adjusted log_2_fold change calculated from p-coefficients extracted from multivariable linear modeling plotted against FDR-corrected p-value. Full results in Suppl. Table 5. **b.** Highlighted box and scatter plots of predicted metabolite abundance by COVID-19 severity. For visualization purposes, technical/true 0s were imputed with a given taxa’s minimum non-zero value prior to log-transformation. Boxes represent medians and interquartile ranges, while whiskers represent 95%ile.

We next sought to evaluate whether changes in microbial communities affected local metabolite production. Using a validated computational workflow to generate putative metabolic profiles from stool metagenomes^29^ (Methods), we found 57 of 80 well-predicted known stool metabolites to be differentially abundant based on COVID-19 disease severity (all FDR-corrected *p*-value < 0.05; Fig. 5a & Suppl. Table 5). We identified the perturbation of bile acid metabolism in severe COVID-19, with relative enrichment of primary bile acids (chenodeoxycholate, cholate, and ketodeoxycholate) alongside depletion of secondary bile acids (lithocholate, lithocholic acid, and deoxycholic acid) (Fig. 5b). Similar to our microbial pathway analysis which revealed reductions in MetaCyc pathways related to SCFA production, predicted levels of butyrate, isobutyrate, and propionate were also reduced in severe COVID-19 (Suppl. Table 5). Furthermore, we confirmed prior data showing relative enrichment of bilirubin^30^, creatine and polyamines (e.g., acetyl-spermidine^31^), and pantothenic acid^32^ in severe COVID-19, as well as a relative depletion of deoxyinosine^32^ (Suppl. Table 5).

## Discussion

In a large U.S. hospital-based cohort of diverse patients admitted with confirmed COVID-19 during the initial year of the pandemic, we found community- and species-level alterations linked to disease severity. Using a random forest machine learner, these microbial features could accurately classify patients based on disease severity, indicating that specific gut microbial configurations may predict a more severe disease course. Network analyses identified significant disruptions to gut ecologic topology in severe COVID-19. Differential abundance testing of microbial pathways and predicted stool metabolites suggest that these disruptions may change the balance of bile acids and SCFAs in the gut, identifying novel treatment opportunities that may ameliorate the severity of COVID-19. We also found significant depletions of two microbes previously associated with long COVID, suggesting early gut microbial disturbances may precede the development of a long-term complication.

Determining who will require a higher level of care remains one of the most challenging questions facing clinicians caring for patients with COVID-19. Our machine learning algorithm demonstrated excellent discrimination between moderate and severe COVID-19 using only gut microbial features. Notably, the inclusion of clinical data did not significantly improve the classification accuracy of our model. Prior work has incorporated such information from initial presentation^33^, multi-cytokine panels^34^, and previously validated illness severity scores^35^ to forecast whether a given patient will suffer from a more severe COVID-19 course. However, based on their performance characteristics, these approaches appear to be less predictive than our microbiome-centered approach.

Our findings expand on prior research linking changes in gut microbial ecology to COVID-19. However, it should be noted that much of the initial work has been done on a smaller scale^7, 9–11,14^ and typically outside of North America^7–15^, limiting their generalizability. Further, these comparative analyses may have focused on specialized populations, such as the very young, the asymptomatic, or patients in recovery^12,16–18^, and may not have been well-suited to consider clinical factors that may confound the relationship between gut microbial communities and COVID-19 using more robust multivariable approaches^7,8,10–17^. Prior studies also predominantly relied on 16S rRNA sequencing to demonstrate community- or genus-level shifts related to COVID-19^7,14–17^, falling short of the species-level resolution and biochemical insights gained by employing next-generation sequencing of gut metagenomes and other functional multi-omic technologies. In contrast, we assembled a large, representative North American patient population admitted with symptomatic COVID-19 whose gut microbial communities were interrogated using metagenomic techniques, allowing us to identify novel microbial features to more comprehensively characterize disease severity with high predictive accuracy.

Prior investigations have observed similar community- and taxa-level alterations in microbial composition in COVID-19. In the earliest phase of the pandemic, a study from Hong Kong (*n* = 36) also demonstrated relative reductions in the group Eubacterium among the gut metagenomes of COVID-19-infected patients compared to referent populations, and like our work, found widespread depletion of typical gut colonizers such as *Faecalibacterium* and *Roseburia* spp. in severe COVID-19^9^. In an expanded population of 100 patients, the same group reaffirmed a reduction in diversity and a loss of health-associated gut commensals in severe COVID-19^13^. Finally, a study of 30 SARS-CoV-2 infected patients in mainland China using 16S rRNA-based sequencing similarly demonstrated a change in gut community structure with reductions in α-diversity compared to referent counterparts^14^. Notably, they also achieved success in classifying stool samples from patients with COVID-19 compared to those from healthy controls or those infected with influenza, indicating the relatively distinct gut ecology of COVID-19. However, their classification tasks were conducted in a smaller population using supervised feature selection (i.e., the top results from their linear discriminant analysis) of genus-level taxa, and arguably, the role of a gut microbial biomarker in discriminating COVID-19 from non-infected individuals is uncertain now that SARS-CoV-2 testing is more widely available^36^.

Our work offers insights beyond these broad characterizations of the gut microbiome in COVID-19. It is appreciated that gut microbial ecology influences the host immune response to viral respiratory infections^3–6^. Our identification of *Blautia wexlerae* and *Eubacterium hallii* as network hubs depleted in severe COVID-19 (both Lachnospiraceae implicated in other immune-mediated diseases^37^) suggests these bacteria may engage in important roles in the regulation of immunity to SARS-CoV-2. Predicted depletion of secondary bile acids in severe disease provides another mechanism by which changes in gut microbial communities may influence the immune response to SARS-CoV-2. Bile acids regulate mucosal and systemic immunity in several ways^38^. Prior work has suggested that secondary bile acids are the primary ligand for TGR5^39^ through which they may suppress pro-inflammatory signaling^38,40^, resulting in impaired immunity to viral infections^41,42^. The predicted shift in bile acid pools may also result in increased regulation of bile acid-sensitive transcription factors, as increased primary bile acids will preferentially activate farsenoid X Receptor, while depletions in secondary bile acids will reduce activation of vitamin D receptor (VDR)^43,44^ and pregnane X receptor (PXR)^45^. Decreased VDR/PXR signaling during active infection are associated with increased systemic inflammation and increased morbidity and mortality^46,47^, possibly contributing to the clinical milieu observed in severe COVID-19. This is a particularly noteworthy hypothesis given emerging epidemiologic data on the link between diet^48^, vitamin D status^49^, and COVID-19 disease risk and severity, as well as early work linking depletion of secondary bile acids to COVID-19-related mortality^50^.

Our study has several key strengths. First, we assembled a large representative cohort of patients at a U.S.-based tertiary care center for whom we collected relevant clinical metadata to complement serial stool sampling. Second, our computational workflow allowed us to not only link community-level changes in gut microbial ecology but species-resolved signatures of severe COVID-19. Third, network analyses identified critical taxa central to maintaining a fragile gut microbial configuration less likely to be found in severe COVID-19, and complementary MetaCyc pathway and predicted metabolite analyses further link these changes to alterations in bile acid pool and SCFA levels. Taken together, these observations serve as proof of principle that using NGS to interrogate gut microbial ecology may generate tractable hypotheses to be explored in follow-up investigations. Finally, our results fit well in the context of independent works from other groups–lending credence to our findings–and using a machine learning classifier, we demonstrate excellent accuracy in discriminating samples from moderate vs. severe COVID-19. These findings hint at the possibility that modulating gut microbial communities may be a viable disease prevention or therapeutic strategy in COVID-19.

We acknowledge several limitations. We were not positioned to assess whether findings differed on the basis of SARS-CoV-2 strain or variants. Our study enrolled patients from April 2020 to May 2021 during which genomic surveillance infrastructure in the U.S. was not equipped to comprehensively explore this question. Prior to the Delta variant wave beginning in June 2021, the majority of COVID-19 cases were either Alpha or other less consequential variants of interest^51^. Given the observational nature of our study, we cannot exclude the possibility of residual confounding. However, we adjusted for multiple potential confounders. All enrolled patients were hospitalized, which may minimize study heterogeneity at the expense of overall generalizability. We also assessed the gut microbiome at the earliest feasible time point on admission. This resulted in variation in the timing of collection, which limits our ability to infer causality. Despite these limitations, our findings are intended to be hypothesis-generating to inform the continuum of research that may logically follow.

Leveraging the gut microbiome as a potential biomarker for disease severity and modulating this fragile ecology to improve COVID-19 outcomes each hold significant appeal in the fight to end this pandemic. Multidisciplinary approaches will be needed to confirm our early findings. Validation of a non-invasive indicator predictive of disease severity could readily identify and target at-risk individuals for more aggressive therapy. Finally, directed probiotic restoration or targeted depletion of severe COVID-19-linked microbes could offer a novel therapeutic modality to complement existing therapies.

## Methods

### Study population

Patients were screened daily for inclusion from among all admitted individuals for whom a designation of possible SARS-CoV-2 infection was flagged by hospital infection control. COVID-19 infection status was subsequently confirmed with at least one positive nasopharyngeal SARS-CoV-2 polymerase chain reaction (PCR) test. An optional biospecimen collection protocol was nested within this longitudinal study, which allowed collection of additional clinically relevant biospecimens, including stool samples.

### Sample/data collection

Fresh stool was collected and refrigerated at 4°C until aliquoting/freezing at −80°C (typically within 4 hours of collection) from adult patients enrolled in the prospective biospecimen collection study. Participants were able to provide stool samples as frequently as once daily, as well as declining donation on any given day (while remaining in the study). Study coordinators blinded to case status abstracted data from the electronic health record using a double data entry approach with discrepancies adjudicated by re-abstraction or after discussions with supervising authors. We collected information on admission age (years), biological sex (male, female, other), race (White, Black, Asian, American Indian, Mixed, or Other), ethnicity (non-Hispanic or Hispanic), admission BMI (kg/m^2^), comorbidities including history of cancer, pulmonary, or cardiac disease, hypertension, hyperlipidemia, and diabetes mellitus (each yes/no), smoking history (active, former, never, unknown, and pack-years among smokers), and their composite admission Charlson Comorbiditiy Index, a validated score predictive of in-hospital mortality^52^. Information on hospital course, including admission Simplified Acute Physiology Score II (SAPS II)^22^ and Sequential Organ Failure Assessment (SOFA) scores^23^ were calculated from routine laboratory results and clinical assessments. The use of antibiotics, antivirals including remdesivir, hydroxychloroquine, corticosteroids, anti-IL-6 therapy, any form of oxygen support, high-flow oxygen, bilevel positive airway pressure (BiPAP) ventilation, or mechanical ventilation (each yes/no) was collected. Mortality within 90 days of admission was ascertained in the post-study period.

### Extraction protocols

Stool samples, reagent-only negative controls, and mock community positive controls (Zymo Research) were extracted using either the AllPrep PowerFecal DNA/RNA 96 Kit (Qiagen) or the Maxwell HT 96 gDNA Blood Isolation System (Promega)^53^. SARS-CoV-2 viral load was quantified as per CDC guidelines^54^ using the 2019-nCoV N1 primer and probe set^54^, as well as human RNaseP as an internal control. Each RT-qPCR reaction contained TaqPath™ 1-Step RT-qPCR Master Mix (Thermo Fisher), RNA template, the CDC N1 or RNaseP forward and reverse primers (IDT), probe, and RNase-free water to a total reaction volume of 10 μl. Viral copy numbers were quantified using N1 quantitative PCR (qPCR) standards (IDT) in 10-fold dilutions to generate a standard curve. The assay was run in triplicate for each sample with three no-template control wells per 384 well plate.

### Microbial Sequencing

Samples were sequenced by two metagenomic sequencing facilities at the Broad Institute and Baylor College of Medicine according to their standard established platforms. DNA was prepared for sequencing using the Illumina Nextera XT DNA library preparation kit. All libraries were sequenced with a target of 3GB output at 2×150bp read length using the Illumina NovaSeq platform. No major batch effects attributable to sequencing center were observed, and thus, subsequent analyses were conducted on pooled samples (multivariable PERMANOVA R^2^ for batch = 1.2%, *p*-value = 0.12, Suppl. Figure 4).

### Sequence bioinformatics

Taxonomic and functional profiles were generated using the bioBakery 3 shotgun metagenome workflow 3.0.0, the details of which have previously been described^55^. Briefly, human reads were filtered using KneadData 0.10.0 and taxonomic profiles generated using MetaPhlAn 3.0.0^56^. Functional profiling was conducted using HUMAnN 3.0.0^56^, resulting in gene family abundance tables assembled into higher order MetaCyc pathways^57^.

Given the tight coupling and relatively conserved nature of gut taxonomic and metabolite profiles^58^, we used the MelonnPan-predict 0.99.023 workflow^29^ to interrogate the functional relationship between COVID-19 severity and microbial community metabolism. In brief, MelonnPan uses an elastic net model to conservatively predict putative metabolite levels based on stool UniRef90 gene family abundance.

### Statistical Analysis

To compare patient characteristics between study groups, we used standard statistical tests, including chi-squared (χ^2^) tests or Fisher’s exact testing for categorical variables, the Student’s *t*-test for normally distributed, non-categorical variables and nonparametric Wilcoxon rank sum tests for all others. Differences with two-tailed *p*-value ≤ 0.05 were considered significant.

α-diversity was calculated using the Shannon index with the “diversity” function from the R package vegan. Principal coordinates analyses (PCoA) were performed using species-level Bray-Curtis dissimilarity metrics with the “vegdist” function in the vegan package.

After filtering out features with no variance and low (< 10%) prevalence, we performed differential abundance testing of species-level taxonomy, MetaCyc pathways, and predicted stool metabolites using linear mixed-effects models to account for a nested data structure from repeated sampling:

log(feature) ~ intercept + COVID-19 severity + age + sex + prior antibiotic use + race + ethnicity + BMI + Charlson Comorbidity Index + remdesivir + corticosteroids + days since admission + SARS-CoV-2 stool viral load + sequencing depth + (1 | participant)

Machine learning model building and evaluation were conducted using the SIAMCAT v.1.13.3 package^59^. Log-transformed species with pseudocount were filtered to remove biomarkers with low overall abundance and z-transformed. A nested cross-validation procedure was applied to calculate prediction accuracy by splitting data into training and testing sets for twice-repeated, five-fold-cross-validation. To account for longitudinal sampling^59^, data splits were stratified by participant ID, ensuring samples from the same individual were used in the same fold. For each split, a random forest (RF) regressor was trained and subsequently used to predict COVID-19 disease severity. To evaluate model performance, we used the lambda parameter to maximize the area under the receiver operator characteristic curve (AUROC) with a 95% confidence interval (CI) for cross-validation error.

To assess whether ecological dynamics may help explain observed differences in taxonomy, we performed dedicated microbial network analyses. To account for our longitudinal data structure and to avoid overfitting, we restricted this analysis to each participant’s first collected stool. Network construction was conducted using the “netConstruct” function in NetCoMi v.1.0.2^60^, normalized using a modified centered-log ratio and limited the resulting network to microbes with an absolute Pearson correlation ≥ 0.4 (approximately equal to the 95th percentile of correlation matrix distribution). Network hubs were identified as those in the top quintile of degree, betweenness, and closeness centrality in each network (moderate vs. severe COVID-19, respectively). Finally, comparison of moderate and severe networks was performed using the “netCompare” function with 10,000 permutations.

### Regulatory compliance and data availability

Study protocols were approved by the Mass General Brigham Institutional Review Board. Study enrollment with written informed consent was conducted with the patient or their healthcare proxy. Prior to publication, raw sequencing data will be deposited at the National Center for Biotechnology Information’s (NCBI) Sequence Read Archive (SRA) under a to-be-determined BioProject accession ID.

## Supplementary Material

Supplement 1
