# Supplementary material for: Metagenomic assessment of gut microbial communities and risk of severe COVID-19
Source: Res Sq. 2022 Jun 7:rs.3.rs-1717624. Preprint. [Version 1] doi: 10.21203/rs.3.rs-1717624/v1 (PMC9176657; doi:10.21203/rs.3.rs-1717624/v1)
Supplement: Supplement 1 [file 2022_0531_covid-stool-2-supplementary-appendix.docx]

# Metagenomic assessment of gut microbial communities and risk of severe COVID-19

Long H. Nguyen^1,2,3^*, Daniel Okin^4^*, David A. Drew^1,2^*, Vincent M. Battista^1,2^, Sirus Jesudasen^5^, Thomas M. Kuntz^3^, Amrisha Bhosle^3,6,7^, Kelsey N. Thompson^3^, Trenton Reinicke^1,2^, Chun-Han Lo^1,2^, Jacqueline E. Woo^1,2^, Alexander Caraballo^1,2^, Lorenzo Berra^8^, Jacob Vieira^4^, Ching-Ying Huang^4^, Upasana Das Adhikari^9^, Minsik Kim^4^, Hui-Yu Sui^4^, Marina Magicheva-Gupta^1,2^, Lauren McIver^7^, Marcia B. Goldberg^10,11,12^, Douglas S. Kwon^9,10^, Curtis Huttenhower^3,6,7,11^, Andrew T. Chan^1,2,3,11^*, Peggy S. Lai^2,4^*

# Supplementary Appendix

**Supplementary Table 1. Participant characteristics**

**Supplementary Table 2. Multivariable linear modeling results (taxonomy)**

**Supplementary Table 3. Multivariable linear modeling results (MetaCyc pathways)**

**Supplementary Table 4. Node and network-specific information**

**Supplementary Table 5. Multivariable linear modeling results (predicted stool metabolites)**

**Supplementary Figure 1. Volcano plot for multivariable linear modeling results (MetaCyc pathways)**

**Supplementary Figure 2. Entropy heatmap for clinical covariates**

**Supplementary Figure 3. Node map**

**Supplementary Figure 4. PCoA by batch**

**Supplementary Table 1. Participant characteristics.**

|  | **COVID-19 Severity** | |  |
| --- | --- | --- | --- |
|  | **Moderate** | **Severe** | ***p*-value** |
| **n** | 48 | 79 |  |
| **Age (mean (SD))** | 58.6 (12.8) | 61.0 (14.4) | 0.34 |
| **Male (%)** | 23 (47.9) | 49 (62.0) | 0.17 |
| **Race (%)** |  |  |  |
| **White** | 32 (66.7) | 48 (61.5) | 0.64 |
| **Black** | 5 (10.4) | 12 (15.4) |  |
| **Asian** | 1 (2.1) | 3 (3.8) |  |
| **American Indian** | 1 (2.1) | 0 (0.0) |  |
| **Mixed** | 1 (2.1) | 4 (5.1) |  |
| **Other** | 8 (16.7) | 11 (14.1) |  |
| **Ethnicity (%)** |  |  | 0.73 |
| **Not Hispanic** | 33 (73.3) | 50 (68.5) |  |
| **Hispanic** | 12 (26.7) | 23 (31.5) |  |
| **Body Mass Index (BMI) (mean kg/m^2^ (SD))** | 30.0 (7.7) | 33.3 (7.5) | 0.02 |
| **Comorbidities** |  |  |  |
| **Charlson Comorbidity Index (mean (SD))** | 3.0 (2.4) | 3.5 (2.4) | 0.24 |
| **Immunosuppression (%)** | 10 (20.8) | 15 (19.0) | 0.98 |
| **Cancer (%)** | 6 (12.5) | 13 (16.5) | 0.73 |
| **Pulmonary (%)** | 19 (39.6) | 30 (38.0) | 1 |
| **Cardiac (%)** | 30 (62.5) | 57 (72.2) | 0.35 |
| **Hypertension (%)** | 20 (41.7) | 47 (59.5) | 0.08 |
| **Hyperlipidemia (%)** | 16 (33.3) | 26 (32.9) | 1 |
| **Diabetes Mellitus (%)** | 10 (20.8) | 30 (38.0) | 0.07 |
| **Smoking (%)** |  |  | 0.52 |
| **Active** | 3 (6.2) | 2 (2.5) |  |
| **Former** | 17 (35.4) | 22 (27.8) |  |
| **Never** | 24 (50.0) | 46 (58.2) |  |
| **Unknown** | 4 (8.3) | 9 (11.4) |  |
| **Pack-Years among active/former (mean (SD))** | 27.7 (24.7) | 24.5 (17.1) | 0.71 |
| **Hospital Course** |  |  |  |
| **Admission SAP Score (mean (SD))** | 22.2 (9.2) | 32.0 (12.5) | <0.001 |
| **Admission SOFA Score (mean (SD))** | 2.7 (1.4) | 5.4 (2.6) | <0.001 |
| **ICU Admission (%)** | 0 (0.0) | 78 (98.7) | <0.001 |
| **Antibiotics (%)** | 14 (29.2) | 39 (49.4) | 0.04 |
| **Antiviral Therapy (%)** | 31 (64.6) | 65 (82.3) | 0.04 |
| **Remdesivir (%)** | 30 (62.5) | 48 (60.8) | 0.99 |
| **Hydroxychloroquine (%)** | 1 (2.1) | 16 (20.3) | 0.01 |
| **Corticosteroids (%)** | 33 (68.8) | 53 (67.1) | 1 |
| **Anti-IL-6 Therapy (%)** | 1 (2.1) | 7 (8.9) | 0.25 |
| **Oxygen Therapy (%)** | 39 (81.2) | 78 (98.7) | 0.001 |
| **High Flow Oxygen (%)** | 0 (0.0) | 39 (49.4) | <0.001 |
| **BPAP (%)** | 0 (0.0) | 13 (16.5) | 0.008 |
| **Mechanical Ventilation (%)** | 0 (0.0) | 74 (93.7) | <0.001 |
| **Deceased within 90 days (%)** | 2 (4.2) | 18 (22.8) | 0.01 |
| Abbreviations: BMI (body mass index), BPAP (bilevel positive airway pressure), ICU (intensive care unit), IL-6 (interleukin-6), kg (kilogram), m (meter), SAP (Simplified Acute Physiology), SD (standard deviation), SOFA (Sequential Organ Failure Assessment) | | | |

**Supplementary Table 2. Multivariable linear modeling results (taxonomy)**

| **feature** | **metadata** | **value** | **coef** | **stderr** | **N** | **N.not.0** | **pval** | **qval** |
| --- | --- | --- | --- | --- | --- | --- | --- | --- |
| *Eubacterium_eligens* | covid_severity | Severe | -5.58 | 0.81 | 241 | 48 | 3.53E-10 | 5.15E-07 |
| *Eubacterium_hallii* | covid_severity | Severe | -5.02 | 0.74 | 241 | 80 | 4.05E-10 | 5.15E-07 |
| *Roseburia_hominis* | covid_severity | Severe | -5.87 | 0.87 | 241 | 48 | 7.31E-10 | 6.20E-07 |
| *Anaerostipes_hadrus* | covid_severity | Severe | -5.11 | 0.79 | 241 | 62 | 1.89E-09 | 1.20E-06 |
| *Fusicatenibacter_saccharivorans* | covid_severity | Severe | -5.66 | 0.88 | 241 | 63 | 2.97E-09 | 1.51E-06 |
| *Adlercreutzia_equolifaciens* | covid_severity | Severe | -5.04 | 0.79 | 241 | 60 | 4.39E-09 | 1.86E-06 |
| *Actinomyces_odontolyticus* | covid_severity | Severe | -2.44 | 0.38 | 241 | 52 | 5.50E-09 | 1.86E-06 |
| *Agathobaculum_butyriciproducens* | covid_severity | Severe | -3.49 | 0.56 | 241 | 55 | 5.86E-09 | 1.86E-06 |
| *Asaccharobacter_celatus* | covid_severity | Severe | -5.42 | 0.87 | 241 | 61 | 6.82E-09 | 1.93E-06 |
| *Clostridium_leptum* | covid_severity | Severe | -3.89 | 0.66 | 241 | 91 | 2.88E-08 | 7.33E-06 |
| *Roseburia_intestinalis* | covid_severity | Severe | -4.03 | 0.69 | 241 | 32 | 5.87E-08 | 1.36E-05 |
| *Blautia_wexlerae* | covid_severity | Severe | -5.00 | 0.88 | 241 | 96 | 7.77E-08 | 1.65E-05 |
| *Streptococcus_parasanguinis* | covid_severity | Severe | -4.17 | 0.73 | 241 | 78 | 8.82E-08 | 1.73E-05 |
| *Dorea_formicigenerans* | covid_severity | Severe | -4.37 | 0.80 | 241 | 56 | 2.56E-07 | 4.65E-05 |
| *Eubacterium_siraeum* | covid_severity | Severe | -4.41 | 0.81 | 241 | 45 | 2.90E-07 | 4.91E-05 |
| *Gemmiger_formicilis* | covid_severity | Severe | -3.93 | 0.72 | 241 | 49 | 3.15E-07 | 5.00E-05 |
| *Enterorhabdus_caecimuris* | covid_severity | Severe | -2.55 | 0.48 | 241 | 56 | 5.26E-07 | 7.87E-05 |
| *Eubacterium_rectale* | covid_severity | Severe | -4.37 | 0.84 | 241 | 55 | 8.59E-07 | 0.000121398 |
| *Actinomyces_sp_HMSC035G02* | covid_severity | Severe | -1.96 | 0.37 | 241 | 37 | 9.54E-07 | 0.000127756 |
| *Oscillibacter_sp_57_20* | covid_severity | Severe | -2.68 | 0.53 | 241 | 31 | 1.34E-06 | 0.000170974 |
| *Ruminococcus_bicirculans* | covid_severity | Severe | -3.53 | 0.70 | 241 | 27 | 2.02E-06 | 0.000244644 |
| *Ruminococcus_torques* | covid_severity | Severe | -3.80 | 0.77 | 241 | 57 | 2.48E-06 | 0.00028714 |
| *Streptococcus_mitis* | covid_severity | Severe | -2.41 | 0.49 | 241 | 29 | 3.21E-06 | 0.000354728 |
| *Blautia_sp_CAG_257* | covid_severity | Severe | -3.29 | 0.68 | 241 | 56 | 3.78E-06 | 0.000400776 |
| *Streptococcus_salivarius* | covid_severity | Severe | -3.18 | 0.66 | 241 | 78 | 4.69E-06 | 0.000477032 |
| *Collinsella_stercoris* | covid_severity | Severe | -2.85 | 0.60 | 241 | 69 | 5.27E-06 | 0.000496993 |
| *Rothia_mucilaginosa* | covid_severity | Severe | -2.53 | 0.53 | 241 | 41 | 5.17E-06 | 0.000496993 |
| *Roseburia_inulinivorans* | covid_severity | Severe | -3.26 | 0.68 | 241 | 46 | 5.54E-06 | 0.00050344 |
| *Streptococcus_salivarius* | time_from_admit | time_from_admit | -0.07 | 0.02 | 241 | 78 | 1.47E-05 | 0.001288644 |
| *Faecalibacterium_prausnitzii* | time_from_admit | time_from_admit | -0.11 | 0.02 | 241 | 110 | 1.59E-05 | 0.001347113 |
| *Actinomyces_sp_HPA0247* | covid_severity | Severe | -1.80 | 0.40 | 241 | 34 | 1.75E-05 | 0.001436743 |
| *Streptococcus_gordonii* | covid_severity | Severe | -1.74 | 0.40 | 241 | 34 | 2.80E-05 | 0.002229106 |
| *Streptococcus_oralis* | covid_severity | Severe | -2.06 | 0.48 | 241 | 36 | 3.45E-05 | 0.002655947 |
| *Monoglobus_pectinilyticus* | covid_severity | Severe | -2.78 | 0.66 | 241 | 31 | 4.45E-05 | 0.003327227 |
| *Firmicutes_bacterium_CAG_145* | stool_viral_avg | stool_viral_avg | 0.98 | 0.24 | 241 | 58 | 4.86E-05 | 0.003501013 |
| *Streptococcus_thermophilus* | covid_severity | Severe | -2.94 | 0.70 | 241 | 70 | 4.95E-05 | 0.003501013 |
| *Bacteroides_ovatus* | read_depth | read_depth | 1.57 | 0.38 | 241 | 153 | 5.43E-05 | 0.003736829 |
| *Actinomyces_oris* | covid_severity | Severe | -1.85 | 0.45 | 241 | 49 | 7.38E-05 | 0.004941991 |
| *Slackia_isoflavoniconvertens* | charlson | charlson | 1.32 | 0.32 | 241 | 29 | 8.36E-05 | 0.005456455 |
| *Clostridium_sp_CAG_58* | covid_severity | Severe | -2.67 | 0.67 | 241 | 36 | 9.81E-05 | 0.006237965 |
| *Dorea_longicatena* | covid_severity | Severe | -3.51 | 0.88 | 241 | 67 | 0.000101332 | 0.006287522 |
| *Bilophila_wadsworthia* | stool_viral_avg | stool_viral_avg | 0.96 | 0.25 | 241 | 83 | 0.000131041 | 0.007937357 |
| *Collinsella_aerofaciens* | covid_severity | Severe | -4.71 | 1.22 | 241 | 105 | 0.000169451 | 0.010025208 |
| *Clostridium_clostridioforme* | male | yes | -2.79 | 0.72 | 241 | 71 | 0.000180928 | 0.010228468 |
| *Coprococcus_catus* | covid_severity | Severe | -2.72 | 0.70 | 241 | 45 | 0.00017905 | 0.010228468 |
| *Eubacterium_eligens* | on_abx | TRUE | -3.00 | 0.78 | 241 | 48 | 0.000227243 | 0.012567528 |
| *Blautia_obeum* | covid_severity | Severe | -3.29 | 0.87 | 241 | 98 | 0.000246471 | 0.013340905 |
| *Roseburia_hominis* | stool_viral_avg | stool_viral_avg | 0.94 | 0.25 | 241 | 48 | 0.000263777 | 0.013980192 |
| *Eubacterium_rectale* | stool_viral_avg | stool_viral_avg | 0.82 | 0.22 | 241 | 55 | 0.000296811 | 0.01540994 |
| *Ruminococcus_gnavus* | male | yes | -3.85 | 1.03 | 241 | 101 | 0.000317686 | 0.016163838 |
| *Methanobrevibacter_smithii* | stool_viral_avg | stool_viral_avg | 1.08 | 0.30 | 241 | 81 | 0.00034851 | 0.017384503 |
| *Roseburia_faecis* | covid_severity | Severe | -2.58 | 0.70 | 241 | 36 | 0.000369361 | 0.018070286 |
| *Bifidobacterium_adolescentis* | covid_severity | Severe | -3.27 | 0.90 | 241 | 37 | 0.000405558 | 0.019286922 |
| *Christensenella_minuta* | stool_viral_avg | stool_viral_avg | 1.04 | 0.29 | 241 | 69 | 0.000409392 | 0.019286922 |
| *Prevotella_copri* | ethnicity | Hispanic | 3.91 | 1.07 | 241 | 45 | 0.000440384 | 0.020369764 |
| *Bacteroides_massiliensis* | tx_steroids | 1 | -4.04 | 1.12 | 241 | 56 | 0.000483858 | 0.021981 |
| *Eubacterium_ramulus* | covid_severity | Severe | -2.08 | 0.58 | 241 | 43 | 0.000526903 | 0.023516517 |
| *Candida_albicans* | covid_severity | Severe | 2.85 | 0.81 | 241 | 58 | 0.000559996 | 0.024562563 |
| *Ruminococcus_gnavus* | covid_severity | Severe | -4.35 | 1.24 | 241 | 101 | 0.000603029 | 0.026001779 |
| *Ruminococcus_bromii* | covid_severity | Severe | -3.75 | 1.09 | 241 | 41 | 0.000810607 | 0.034369741 |
| *Coprococcus_comes* | covid_severity | Severe | -2.88 | 0.85 | 241 | 58 | 0.000985043 | 0.041081154 |
| *Alistipes_putredinis* | stool_viral_avg | stool_viral_avg | 0.88 | 0.26 | 241 | 116 | 0.001031779 | 0.042336231 |
| *Enterorhabdus_caecimuris* | read_depth | read_depth | 0.52 | 0.16 | 241 | 56 | 0.001105246 | 0.044630891 |
| *Alistipes_finegoldii* | stool_viral_avg | stool_viral_avg | 0.86 | 0.26 | 241 | 128 | 0.001197463 | 0.047599135 |
| *Faecalicatena_orotica* | time_from_admit | time_from_admit | 0.06 | 0.02 | 241 | 33 | 0.001248359 | 0.048858837 |
| *Clostridium_symbiosum* | read_depth | read_depth | 0.97 | 0.30 | 241 | 81 | 0.001283603 | 0.049477043 |
| *Bifidobacterium_adolescentis* | on_abx | TRUE | -2.87 | 0.87 | 241 | 37 | 0.001330828 | 0.049644289 |
| *Blautia_hydrogenotrophica* | covid_severity | Severe | -2.98 | 0.91 | 241 | 79 | 0.00135305 | 0.049644289 |
| *Lactococcus_lactis* | covid_severity | Severe | -1.18 | 0.36 | 241 | 30 | 0.001351994 | 0.049644289 |
| *Slackia_isoflavoniconvertens* | age | age | -1.05 | 0.32 | 241 | 29 | 0.001365999 | 0.049644289 |
| *Bacteroides_stercoris* | read_depth | read_depth | 0.96 | 0.30 | 241 | 100 | 0.001496726 | 0.052361287 |
| *Dorea_sp_CAG_317* | covid_severity | Severe | -2.17 | 0.67 | 241 | 39 | 0.001469326 | 0.052361287 |
| *Roseburia_faecis* | on_abx | TRUE | -2.23 | 0.69 | 241 | 36 | 0.001502505 | 0.052361287 |
| *Enterococcus_faecalis* | time_from_admit | time_from_admit | 0.07 | 0.02 | 241 | 117 | 0.001537576 | 0.052859367 |
| *Staphylococcus_epidermidis* | time_from_admit | time_from_admit | 0.04 | 0.01 | 241 | 30 | 0.001564179 | 0.053056953 |
| *Enterococcus_faecium* | time_from_admit | time_from_admit | 0.08 | 0.03 | 241 | 85 | 0.001588931 | 0.053187362 |
| *Erysipelatoclostridium_ramosum* | stool_viral_avg | stool_viral_avg | 1.04 | 0.33 | 241 | 103 | 0.001711215 | 0.056536756 |
| *Bifidobacterium_longum* | covid_severity | Severe | -3.86 | 1.21 | 241 | 78 | 0.001744827 | 0.056908195 |
| *Streptococcus_salivarius* | stool_viral_avg | stool_viral_avg | 0.68 | 0.21 | 241 | 78 | 0.001777462 | 0.05723879 |
| *Eubacterium_eligens* | tx_remdesivir | 1 | 2.94 | 0.92 | 241 | 48 | 0.001864039 | 0.059276445 |
| *Clostridium_spiroforme* | covid_severity | Severe | -1.66 | 0.53 | 241 | 32 | 0.002000588 | 0.062833294 |
| *Actinomyces_sp_HPA0247* | age | age | 0.61 | 0.19 | 241 | 34 | 0.00205807 | 0.06385037 |
| *Alistipes_onderdonkii* | stool_viral_avg | stool_viral_avg | 0.25 | 0.08 | 241 | 27 | 0.002182969 | 0.066909322 |
| *Akkermansia_muciniphila* | tx_remdesivir | 1 | 4.34 | 1.39 | 241 | 65 | 0.002259041 | 0.068416668 |
| *Alistipes_putredinis* | tx_remdesivir | 1 | 3.73 | 1.20 | 241 | 116 | 0.002290425 | 0.068551062 |
| *Clostridium_scindens* | read_depth | read_depth | 0.88 | 0.29 | 241 | 82 | 0.002374711 | 0.070247255 |
| *Faecalibacterium_prausnitzii* | covid_severity | Severe | -3.22 | 1.05 | 241 | 110 | 0.002619834 | 0.076607573 |
| *Roseburia_inulinivorans* | stool_viral_avg | stool_viral_avg | 0.67 | 0.22 | 241 | 46 | 0.002781937 | 0.08042328 |
| *Anaerotruncus_colihominis* | stool_viral_avg | stool_viral_avg | 0.82 | 0.27 | 241 | 79 | 0.002854961 | 0.080575306 |
| *Bifidobacterium_adolescentis* | race | Other | -4.55 | 1.49 | 241 | 37 | 0.002913887 | 0.080575306 |
| *Collinsella_massiliensis* | covid_severity | Severe | -1.16 | 0.38 | 241 | 48 | 0.002911007 | 0.080575306 |
| *Eubacterium_hallii* | time_from_admit | time_from_admit | -0.06 | 0.02 | 241 | 80 | 0.00284829 | 0.080575306 |
| *Collinsella_aerofaciens* | stool_viral_avg | stool_viral_avg | 1.13 | 0.38 | 241 | 105 | 0.003000269 | 0.082071887 |
| *Clostridium_methylpentosum* | read_depth | read_depth | 0.53 | 0.18 | 241 | 41 | 0.003050523 | 0.082558826 |
| *Enterococcus_faecalis* | covid_severity | Severe | 2.78 | 0.93 | 241 | 117 | 0.003084942 | 0.082611489 |
| *Alistipes_putredinis* | tx_steroids | 1 | -3.75 | 1.24 | 241 | 116 | 0.003148631 | 0.083438709 |
| *Adlercreutzia_equolifaciens* | read_depth | read_depth | 0.70 | 0.23 | 241 | 60 | 0.003211461 | 0.084226354 |
| *Intestinimonas_butyriciproducens* | covid_severity | Severe | -2.58 | 0.87 | 241 | 57 | 0.003478282 | 0.090293352 |
| *Catabacter_hongkongensis* | stool_viral_avg | stool_viral_avg | 0.61 | 0.21 | 241 | 56 | 0.003551759 | 0.090643154 |
| *Streptococcus_mitis* | read_depth | read_depth | 0.50 | 0.17 | 241 | 29 | 0.003563017 | 0.090643154 |
| *Akkermansia_muciniphila* | covid_severity | Severe | -3.61 | 1.22 | 241 | 65 | 0.003657401 | 0.092123044 |
| *Lactobacillus_rhamnosus* | on_abx | TRUE | 2.79 | 0.94 | 241 | 106 | 0.00373657 | 0.093194447 |
| *Asaccharobacter_celatus* | read_depth | read_depth | 0.75 | 0.26 | 241 | 61 | 0.003790563 | 0.09362323 |
| *Pseudomonas_aeruginosa_group* | race | Black | 2.12 | 0.72 | 241 | 26 | 0.003940362 | 0.096387317 |
| *Bifidobacterium_longum* | time_from_admit | time_from_admit | -0.08 | 0.03 | 241 | 78 | 0.004181222 | 0.101305042 |
| *Intestinimonas_butyriciproducens* | stool_viral_avg | stool_viral_avg | 0.73 | 0.25 | 241 | 57 | 0.004256881 | 0.102165132 |
| *Asaccharobacter_celatus* | stool_viral_avg | stool_viral_avg | 0.68 | 0.23 | 241 | 61 | 0.004402636 | 0.104675752 |
| *Bifidobacterium_adolescentis* | male | yes | -2.21 | 0.76 | 241 | 37 | 0.004671986 | 0.106458453 |
| *Coprococcus_catus* | age | age | -1.04 | 0.36 | 241 | 45 | 0.004545777 | 0.106458453 |
| *Coprococcus_comes* | age | age | -1.24 | 0.43 | 241 | 58 | 0.00468685 | 0.106458453 |
| *Parabacteroides_distasonis* | tx_remdesivir | 1 | 3.91 | 1.36 | 241 | 164 | 0.004628298 | 0.106458453 |
| *Roseburia_inulinivorans* | time_from_admit | time_from_admit | -0.05 | 0.02 | 241 | 46 | 0.004661124 | 0.106458453 |
| *Bifidobacterium_longum* | stool_viral_avg | stool_viral_avg | 1.03 | 0.36 | 241 | 78 | 0.005014341 | 0.112043291 |
| *Butyricimonas_synergistica* | age | age | -0.91 | 0.32 | 241 | 37 | 0.005020808 | 0.112043291 |
| *Clostridium_sp_CAG_58* | age | age | -0.92 | 0.32 | 241 | 36 | 0.00525945 | 0.116348175 |
| *Gordonibacter_pamelaeae* | stool_viral_avg | stool_viral_avg | 0.73 | 0.26 | 241 | 164 | 0.005320728 | 0.116689063 |
| *Firmicutes_bacterium_CAG_145* | covid_severity | Severe | -2.48 | 0.88 | 241 | 58 | 0.005381672 | 0.117016866 |
| *Parabacteroides_merdae* | stool_viral_avg | stool_viral_avg | 0.80 | 0.28 | 241 | 126 | 0.005428295 | 0.117030351 |
| *Clostridium_bolteae* | time_from_admit | time_from_admit | -0.06 | 0.02 | 241 | 112 | 0.005583849 | 0.119372375 |
| *Bilophila_wadsworthia* | covid_severity | Severe | -2.33 | 0.83 | 241 | 83 | 0.005738971 | 0.120660689 |
| *Eisenbergiella_tayi* | covid_severity | Severe | -2.72 | 0.97 | 241 | 92 | 0.005708216 | 0.120660689 |
| *Streptococcus_gordonii* | race | American Indian | 5.53 | 1.97 | 241 | 34 | 0.005813717 | 0.121086244 |
| *Streptococcus_oralis* | race | Black | 1.59 | 0.55 | 241 | 36 | 0.005854406 | 0.121086244 |
| *Lactobacillus_rhamnosus* | time_from_admit | time_from_admit | 0.07 | 0.02 | 241 | 106 | 0.006013572 | 0.123375225 |
| *Ruminococcus_bromii* | stool_viral_avg | stool_viral_avg | 0.86 | 0.31 | 241 | 41 | 0.006079994 | 0.123740038 |
| *Alistipes_shahii* | stool_viral_avg | stool_viral_avg | 0.96 | 0.35 | 241 | 106 | 0.00614932 | 0.124157699 |
| *Coprococcus_catus* | on_abx | TRUE | -1.90 | 0.68 | 241 | 45 | 0.006299679 | 0.126191996 |
| *Clostridium_innocuum* | read_depth | read_depth | 1.19 | 0.43 | 241 | 168 | 0.006559481 | 0.129359065 |
| *Dorea_formicigenerans* | time_from_admit | time_from_admit | -0.05 | 0.02 | 241 | 56 | 0.006517488 | 0.129359065 |
| *Bacteroides_vulgatus* | time_from_admit | time_from_admit | -0.06 | 0.02 | 241 | 171 | 0.006642948 | 0.129997386 |
| *Adlercreutzia_equolifaciens* | stool_viral_avg | stool_viral_avg | 0.59 | 0.21 | 241 | 60 | 0.006702226 | 0.130156207 |
| *Clostridium_citroniae* | read_depth | read_depth | 0.81 | 0.30 | 241 | 66 | 0.00709512 | 0.135708076 |
| *Enterococcus_avium* | ethnicity | Hispanic | 2.04 | 0.74 | 241 | 28 | 0.007069445 | 0.135708076 |
| *Lawsonibacter_asaccharolyticus* | read_depth | read_depth | 0.84 | 0.31 | 241 | 103 | 0.007148145 | 0.135708076 |
| *Dorea_formicigenerans* | race | Mixed | -4.88 | 1.78 | 241 | 56 | 0.007255365 | 0.136723332 |
| *Clostridium_scindens* | ethnicity | Hispanic | -2.53 | 0.92 | 241 | 82 | 0.007459094 | 0.139528941 |
| *Klebsiella_variicola* | time_from_admit | time_from_admit | 0.04 | 0.02 | 241 | 26 | 0.007597662 | 0.141083591 |
| *Clostridium_sp_CAG_58* | tx_remdesivir | 1 | 2.03 | 0.75 | 241 | 36 | 0.007672947 | 0.141449106 |
| *Oscillibacter_sp_57_20* | race | Other | 2.41 | 0.89 | 241 | 31 | 0.007729655 | 0.141469363 |
| *Blautia_wexlerae* | time_from_admit | time_from_admit | -0.06 | 0.02 | 241 | 96 | 0.007808002 | 0.141882557 |
| *Agathobaculum_butyriciproducens* | on_abx | TRUE | -1.46 | 0.54 | 241 | 55 | 0.0079358 | 0.1431821 |
| *Butyricimonas_synergistica* | race | American Indian | 8.16 | 3.02 | 241 | 37 | 0.008049207 | 0.143418374 |
| *Rothia_mucilaginosa* | read_depth | read_depth | 0.43 | 0.16 | 241 | 41 | 0.008061646 | 0.143418374 |
| *Bacteroides_intestinalis* | race | American Indian | 8.51 | 3.17 | 241 | 26 | 0.008365406 | 0.147110089 |
| *Fusicatenibacter_saccharivorans* | time_from_admit | time_from_admit | -0.05 | 0.02 | 241 | 63 | 0.008384812 | 0.147110089 |
| *Collinsella_stercoris* | stool_viral_avg | stool_viral_avg | 0.47 | 0.18 | 241 | 69 | 0.008452141 | 0.147275661 |
| *Sellimonas_intestinalis* | stool_viral_avg | stool_viral_avg | 0.84 | 0.32 | 241 | 101 | 0.008674535 | 0.150122563 |
| *Bifidobacterium_adolescentis* | age | age | -1.21 | 0.45 | 241 | 37 | 0.008980572 | 0.154137839 |
| *Streptococcus_vestibularis* | race | American Indian | 7.98 | 3.01 | 241 | 47 | 0.009027727 | 0.154137839 |
| *Eubacterium_rectale* | time_from_admit | time_from_admit | -0.04 | 0.02 | 241 | 55 | 0.009242868 | 0.155720901 |
| *Methanobrevibacter_smithii* | covid_severity | Severe | -2.93 | 1.11 | 241 | 81 | 0.009187788 | 0.155720901 |
| *Rothia_dentocariosa* | race | American Indian | 4.96 | 1.88 | 241 | 28 | 0.009328019 | 0.156121577 |
| *Anaerofustis_stercorihominis* | stool_viral_avg | stool_viral_avg | 0.46 | 0.18 | 241 | 44 | 0.009495802 | 0.157837168 |
| *Staphylococcus_epidermidis* | read_depth | read_depth | -0.53 | 0.20 | 241 | 30 | 0.009554608 | 0.157837168 |
| *Clostridium_leptum* | stool_viral_avg | stool_viral_avg | 0.51 | 0.19 | 241 | 91 | 0.009646923 | 0.158334007 |
| *Bifidobacterium_adolescentis* | time_from_admit | time_from_admit | -0.05 | 0.02 | 241 | 37 | 0.010534492 | 0.170705317 |
| *Parabacteroides_johnsonii* | race | American Indian | 6.91 | 2.66 | 241 | 25 | 0.01053488 | 0.170705317 |
| *Dorea_formicigenerans* | ethnicity | Hispanic | 2.53 | 0.97 | 241 | 56 | 0.010847738 | 0.173563814 |
| *Streptococcus_vestibularis* | stool_viral_avg | stool_viral_avg | 0.52 | 0.20 | 241 | 47 | 0.010813357 | 0.173563814 |
| *Intestinimonas_butyriciproducens* | time_from_admit | time_from_admit | -0.05 | 0.02 | 241 | 57 | 0.011242704 | 0.17875899 |
| *Bacteroides_cellulosilyticus* | time_from_admit | time_from_admit | -0.05 | 0.02 | 241 | 51 | 0.011637323 | 0.183884159 |
| *Anaeromassilibacillus_sp_An250* | charlson | charlson | -0.64 | 0.25 | 241 | 31 | 0.011711613 | 0.183915696 |
| *Acidaminococcus_intestini* | read_depth | read_depth | 0.79 | 0.31 | 241 | 43 | 0.011846146 | 0.184887094 |
| *Agathobaculum_butyriciproducens* | ethnicity | Hispanic | 1.73 | 0.68 | 241 | 55 | 0.012300713 | 0.188160045 |
| *Clostridium_clostridioforme* | tx_remdesivir | 1 | 2.57 | 1.01 | 241 | 71 | 0.012273133 | 0.188160045 |
| *Gordonibacter_pamelaeae* | time_from_admit | time_from_admit | -0.05 | 0.02 | 241 | 164 | 0.012351701 | 0.188160045 |
| *Streptococcus_vestibularis* | time_from_admit | time_from_admit | -0.04 | 0.01 | 241 | 47 | 0.012254755 | 0.188160045 |
| *Akkermansia_muciniphila* | stool_viral_avg | stool_viral_avg | 0.87 | 0.35 | 241 | 65 | 0.012468531 | 0.188590585 |
| *Anaerotruncus_colihominis* | charlson | charlson | 1.02 | 0.40 | 241 | 79 | 0.012750621 | 0.188590585 |
| *Coprococcus_catus* | time_from_admit | time_from_admit | -0.04 | 0.01 | 241 | 45 | 0.012713554 | 0.188590585 |
| *Klebsiella_pneumoniae* | time_from_admit | time_from_admit | 0.04 | 0.01 | 241 | 31 | 0.012643431 | 0.188590585 |
| *Roseburia_inulinivorans* | race | Mixed | -3.80 | 1.49 | 241 | 46 | 0.012665439 | 0.188590585 |
| *Ruthenibacterium_lactatiformans* | read_depth | read_depth | 1.02 | 0.41 | 241 | 183 | 0.013287639 | 0.195397414 |
| *Faecalicatena_orotica* | read_depth | read_depth | 0.66 | 0.27 | 241 | 33 | 0.013598293 | 0.198816425 |
| *Collinsella_aerofaciens* | time_from_admit | time_from_admit | -0.07 | 0.03 | 241 | 105 | 0.013756706 | 0.198846663 |
| *Holdemanella_biformis* | covid_severity | Severe | -1.51 | 0.61 | 241 | 42 | 0.013808989 | 0.198846663 |
| *Staphylococcus_epidermidis* | on_abx | TRUE | -1.06 | 0.42 | 241 | 30 | 0.01383485 | 0.198846663 |
| *Staphylococcus_epidermidis* | race | Black | -1.37 | 0.55 | 241 | 30 | 0.014209952 | 0.202348345 |
| *Streptococcus_parasanguinis* | time_from_admit | time_from_admit | -0.05 | 0.02 | 241 | 78 | 0.01423756 | 0.202348345 |
| *Anaerostipes_hadrus* | time_from_admit | time_from_admit | -0.04 | 0.02 | 241 | 62 | 0.014463476 | 0.204417126 |
| *Rothia_dentocariosa* | covid_severity | Severe | -0.94 | 0.38 | 241 | 28 | 0.014715218 | 0.206826041 |
| *Eisenbergiella_massiliensis* | stool_viral_avg | stool_viral_avg | 0.55 | 0.22 | 241 | 63 | 0.015029637 | 0.20868462 |
| *Eubacterium_eligens* | male | yes | 1.68 | 0.68 | 241 | 48 | 0.015093542 | 0.20868462 |
| *Roseburia_inulinivorans* | on_abx | TRUE | -1.61 | 0.65 | 241 | 46 | 0.01497856 | 0.20868462 |
| *Eubacterium_siraeum* | male | yes | 1.74 | 0.71 | 241 | 45 | 0.015939887 | 0.219194993 |
| *Clostridium_sp_CAG_58* | race | Asian | 3.47 | 1.41 | 241 | 36 | 0.016190282 | 0.221441277 |
| *Blautia_coccoides* | read_depth | read_depth | 0.83 | 0.34 | 241 | 84 | 0.016821983 | 0.225237495 |
| *Gemmiger_formicilis* | age | age | -0.88 | 0.36 | 241 | 49 | 0.016651376 | 0.225237495 |
| *Lawsonibacter_asaccharolyticus* | tx_remdesivir | 1 | 2.33 | 0.96 | 241 | 103 | 0.01680232 | 0.225237495 |
| *Veillonella_parvula* | read_depth | read_depth | 0.78 | 0.32 | 241 | 45 | 0.016787589 | 0.225237495 |
| *Anaerofustis_stercorihominis* | charlson | charlson | 0.71 | 0.29 | 241 | 44 | 0.017119364 | 0.226831579 |
| *Proteobacteria_bacterium_CAG_139* | male | yes | -1.40 | 0.58 | 241 | 57 | 0.017073037 | 0.226831579 |
| *Eubacterium_rectale* | on_abx | TRUE | -1.98 | 0.82 | 241 | 55 | 0.017228755 | 0.227098203 |
| *Clostridium_scindens* | stool_viral_avg | stool_viral_avg | 0.62 | 0.26 | 241 | 82 | 0.017741012 | 0.232645029 |
| *Fusicatenibacter_saccharivorans* | on_abx | TRUE | -2.05 | 0.85 | 241 | 63 | 0.018176371 | 0.237131729 |
| *Odoribacter_splanchnicus* | on_abx | TRUE | -2.18 | 0.91 | 241 | 107 | 0.018525277 | 0.239229977 |
| *Slackia_isoflavoniconvertens* | male | yes | 1.29 | 0.54 | 241 | 29 | 0.018484946 | 0.239229977 |
| *Bifidobacterium_breve* | age | age | -0.92 | 0.38 | 241 | 28 | 0.018953186 | 0.239885098 |
| *Candida_albicans* | read_depth | read_depth | -0.72 | 0.30 | 241 | 58 | 0.018820619 | 0.239885098 |
| *Streptococcus_anginosus_group* | race | American Indian | 8.35 | 3.52 | 241 | 58 | 0.018932468 | 0.239885098 |
| *Veillonella_parvula* | race | American Indian | 10.14 | 4.27 | 241 | 45 | 0.018891172 | 0.239885098 |
| *Bacteroides_cellulosilyticus* | stool_viral_avg | stool_viral_avg | 0.65 | 0.27 | 241 | 51 | 0.019445057 | 0.243685843 |
| *Lactobacillus_reuteri* | covid_severity | Severe | -1.38 | 0.58 | 241 | 27 | 0.019358656 | 0.243685843 |
| *Faecalibacterium_prausnitzii* | on_abx | TRUE | -2.39 | 1.01 | 241 | 110 | 0.019723042 | 0.245957935 |
| *Erysipelatoclostridium_ramosum* | covid_severity | Severe | -2.46 | 1.05 | 241 | 103 | 0.020138427 | 0.249661684 |
| *Turicimonas_muris* | male | yes | -0.99 | 0.42 | 241 | 49 | 0.020216316 | 0.249661684 |
| Results with FDR-corrected p-value <0.25 shown. | | | | | | | | |

**Supplementary Table 3. Multivariable linear modeling results (MetaCyc pathways)**

| **feature** | **metadata** | **value** | **coef** | **stderr** | **N** | **N.not.0** | **pval** | **qval** |
| --- | --- | --- | --- | --- | --- | --- | --- | --- |
| PWY.7046..4.coumarate.degradation..anaerobic. | covid_severity | Severe | -2.69 | 0.45 | 241 | 101 | 1.86E-08 | 0.000120124 |
| PWY.7228..superpathway.of.guanosine.nucleotides.de.novo.biosynthesis.I | covid_severity | Severe | 0.71 | 0.14 | 241 | 241 | 1.59E-06 | 0.005123916 |
| PWY.7663..gondoate.biosynthesis..anaerobic. | covid_severity | Severe | 0.77 | 0.16 | 241 | 241 | 2.60E-06 | 0.005612303 |
| FERMENTATION.PWY..mixed.acid.fermentation | covid_severity | Severe | -2.42 | 0.53 | 241 | 194 | 9.69E-06 | 0.015668685 |
| METH.ACETATE.PWY..methanogenesis.from.acetate | covid_severity | Severe | -2.49 | 0.59 | 241 | 146 | 4.18E-05 | 0.0277281 |
| PWY.5177..glutaryl.CoA.degradation | covid_severity | Severe | -2.22 | 0.53 | 241 | 200 | 4.29E-05 | 0.0277281 |
| PWY.5973..cis.vaccenate.biosynthesis | covid_severity | Severe | 0.67 | 0.15 | 241 | 241 | 2.27E-05 | 0.0277281 |
| PWY.6895..superpathway.of.thiamin.diphosphate.biosynthesis.II | covid_severity | Severe | -2.54 | 0.59 | 241 | 146 | 3.23E-05 | 0.0277281 |
| PWY.7209..superpathway.of.pyrimidine.ribonucleosides.degradation | covid_severity | Severe | -2.32 | 0.53 | 241 | 90 | 2.61E-05 | 0.0277281 |
| PYRIDNUCSAL.PWY..NAD.salvage.pathway.I | covid_severity | Severe | -2.47 | 0.59 | 241 | 172 | 4.15E-05 | 0.0277281 |
| MET.SAM.PWY..superpathway.of.S.adenosyl.L.methionine.biosynthesis | covid_severity | Severe | -1.72 | 0.43 | 241 | 226 | 7.81E-05 | 0.031198869 |
| METSYN.PWY..L.homoserine.and.L.methionine.biosynthesis | covid_severity | Severe | -1.64 | 0.41 | 241 | 226 | 9.02E-05 | 0.031198869 |
| PPGPPMET.PWY..ppGpp.biosynthesis | covid_severity | Severe | -2.74 | 0.67 | 241 | 192 | 7.20E-05 | 0.031198869 |
| PWY.5188..tetrapyrrole.biosynthesis.I..from.glutamate. | covid_severity | Severe | -1.93 | 0.48 | 241 | 226 | 9.61E-05 | 0.031198869 |
| PWY.6125..superpathway.of.guanosine.nucleotides.de.novo.biosynthesis.II | covid_severity | Severe | 0.59 | 0.14 | 241 | 240 | 7.28E-05 | 0.031198869 |
| PWY.6891..thiazole.biosynthesis.II..Bacillus. | covid_severity | Severe | -2.64 | 0.64 | 241 | 146 | 6.69E-05 | 0.031198869 |
| PWY.7003..glycerol.degradation.to.butanol | covid_severity | Severe | -1.91 | 0.48 | 241 | 183 | 9.65E-05 | 0.031198869 |
| PWY.7383..anaerobic.energy.metabolism..invertebrates..cytosol. | covid_severity | Severe | -1.98 | 0.48 | 241 | 201 | 6.47E-05 | 0.031198869 |
| PWY.7392..taxadiene.biosynthesis..engineered. | covid_severity | Severe | -2.76 | 0.68 | 241 | 98 | 8.72E-05 | 0.031198869 |
| PWY66.399..gluconeogenesis.III | covid_severity | Severe | -1.79 | 0.45 | 241 | 199 | 8.81E-05 | 0.031198869 |
| GLYCOGENSYNTH.PWY..glycogen.biosynthesis.I..from.ADP.D.Glucose. | covid_severity | Severe | -1.33 | 0.34 | 241 | 236 | 0.000137264 | 0.034351109 |
| HOMOSER.METSYN.PWY..L.methionine.biosynthesis.I | covid_severity | Severe | -1.72 | 0.44 | 241 | 226 | 0.000124356 | 0.034351109 |
| PWY.5028..L.histidine.degradation.II | time_from_admit | time_from_admit | 0.05 | 0.01 | 241 | 66 | 0.000144811 | 0.034351109 |
| PWY.5347..superpathway.of.L.methionine.biosynthesis..transsulfuration. | covid_severity | Severe | -1.55 | 0.40 | 241 | 226 | 0.000148775 | 0.034351109 |
| PWY.5505..L.glutamate.and.L.glutamine.biosynthesis | covid_severity | Severe | -2.42 | 0.61 | 241 | 177 | 0.000116027 | 0.034351109 |
| PWY.5676..acetyl.CoA.fermentation.to.butanoate.II | covid_severity | Severe | -2.01 | 0.51 | 241 | 202 | 0.000135167 | 0.034351109 |
| PWY.6396..superpathway.of.2.3.butanediol.biosynthesis | charlson | charlson | 0.68 | 0.17 | 241 | 53 | 0.000139923 | 0.034351109 |
| PWY.7115..C4.photosynthetic.carbon.assimilation.cycle..NAD.ME.type | covid_severity | Severe | -2.48 | 0.63 | 241 | 183 | 0.000129138 | 0.034351109 |
| PWY.7456..mannan.degradation | read_depth | read_depth | 0.81 | 0.21 | 241 | 201 | 0.000172108 | 0.038368121 |
| METH.ACETATE.PWY..methanogenesis.from.acetate | time_from_admit | time_from_admit | -0.05 | 0.01 | 241 | 146 | 0.000239688 | 0.04070988 |
| PWY.241..C4.photosynthetic.carbon.assimilation.cycle..NADP.ME.type | covid_severity | Severe | -1.92 | 0.51 | 241 | 214 | 0.000245582 | 0.04070988 |
| PWY.5121..superpathway.of.geranylgeranyl.diphosphate.biosynthesis.II..via.MEP. | covid_severity | Severe | -2.55 | 0.67 | 241 | 101 | 0.000230457 | 0.04070988 |
| PWY.6353..purine.nucleotides.degradation.II..aerobic. | covid_severity | Severe | -1.94 | 0.52 | 241 | 202 | 0.000235579 | 0.04070988 |
| PWY.6549..L.glutamine.biosynthesis.III | covid_severity | Severe | -1.81 | 0.48 | 241 | 200 | 0.00024263 | 0.04070988 |
| PWY.6595..superpathway.of.guanosine.nucleotides.degradation..plants. | read_depth | read_depth | 0.73 | 0.19 | 241 | 209 | 0.000207692 | 0.04070988 |
| PWY.7220..adenosine.deoxyribonucleotides.de.novo.biosynthesis.II | covid_severity | Severe | 0.43 | 0.11 | 241 | 241 | 0.000242783 | 0.04070988 |
| PWY.7222..guanosine.deoxyribonucleotides.de.novo.biosynthesis.II | covid_severity | Severe | 0.43 | 0.11 | 241 | 241 | 0.000242783 | 0.04070988 |
| PWY66.388..fatty.acid..alpha..oxidation.III | race | Black | 1.99 | 0.51 | 241 | 43 | 0.000211707 | 0.04070988 |
| SALVADEHYPOX.PWY..adenosine.nucleotides.degradation.II | covid_severity | Severe | -2.10 | 0.55 | 241 | 203 | 0.000200181 | 0.04070988 |
| PWY.7332..superpathway.of.UDP.N.acetylglucosamine.derived.O.antigen.building.blocks.biosynthesis | read_depth | read_depth | 0.83 | 0.22 | 241 | 160 | 0.000253427 | 0.040960158 |
| PWY.5181..toluene.degradation.III..aerobic...via.p.cresol. | time_from_admit | time_from_admit | 0.04 | 0.01 | 241 | 43 | 0.000333688 | 0.051364185 |
| PWY0.781..aspartate.superpathway | covid_severity | Severe | -2.30 | 0.63 | 241 | 160 | 0.000332312 | 0.051364185 |
| P4.PWY..superpathway.of.L.lysine..L.threonine.and.L.methionine.biosynthesis.I | covid_severity | Severe | -2.33 | 0.64 | 241 | 161 | 0.000344594 | 0.051795018 |
| PWY.5941..glycogen.degradation.II..eukaryotic. | read_depth | read_depth | 0.91 | 0.25 | 241 | 189 | 0.000352511 | 0.051795018 |
| PWY.6588..pyruvate.fermentation.to.acetone | covid_severity | Severe | -2.02 | 0.57 | 241 | 190 | 0.000445671 | 0.064028094 |
| PWY66.409..superpathway.of.purine.nucleotide.salvage | read_depth | read_depth | 0.53 | 0.15 | 241 | 227 | 0.000456243 | 0.064121928 |
| PWY.4984..urea.cycle | read_depth | read_depth | 0.77 | 0.22 | 241 | 225 | 0.000481855 | 0.065100834 |
| PWY.7560..methylerythritol.phosphate.pathway.II | covid_severity | Severe | -2.48 | 0.69 | 241 | 102 | 0.000483347 | 0.065100834 |
| GOLPDLCAT.PWY..superpathway.of.glycerol.degradation.to.1.3.propanediol | covid_severity | Severe | -2.10 | 0.59 | 241 | 205 | 0.000510221 | 0.067317975 |
| PWY0.1297..superpathway.of.purine.deoxyribonucleosides.degradation | time_from_admit | time_from_admit | 0.04 | 0.01 | 241 | 231 | 0.000561158 | 0.072557791 |
| NONMEVIPP.PWY..methylerythritol.phosphate.pathway.I | covid_severity | Severe | -2.72 | 0.77 | 241 | 102 | 0.000615629 | 0.076567719 |
| PWY.6270..isoprene.biosynthesis.I | covid_severity | Severe | -2.86 | 0.82 | 241 | 102 | 0.000615858 | 0.076567719 |
| ARGSYNBSUB.PWY..L.arginine.biosynthesis.II..acetyl.cycle. | covid_severity | Severe | -1.29 | 0.37 | 241 | 237 | 0.000698707 | 0.084102993 |
| PWY.7196..superpathway.of.pyrimidine.ribonucleosides.salvage | covid_severity | Severe | -1.50 | 0.44 | 241 | 205 | 0.000702484 | 0.084102993 |
| P161.PWY..acetylene.degradation | time_from_admit | time_from_admit | 0.05 | 0.01 | 241 | 217 | 0.000734155 | 0.086296632 |
| PWY.7385..1.3.propanediol.biosynthesis..engineered. | time_from_admit | time_from_admit | -0.05 | 0.01 | 241 | 80 | 0.000748387 | 0.086398651 |
| P621.PWY..nylon.6.oligomer.degradation | read_depth | read_depth | 0.92 | 0.27 | 241 | 134 | 0.000774614 | 0.087415743 |
| PWY.7117..C4.photosynthetic.carbon.assimilation.cycle..PEPCK.type | covid_severity | Severe | -1.90 | 0.56 | 241 | 214 | 0.000795859 | 0.087415743 |
| PWY.7312..dTDP.D..beta..fucofuranose.biosynthesis | read_depth | read_depth | 0.66 | 0.19 | 241 | 91 | 0.000797762 | 0.087415743 |
| P461.PWY..hexitol.fermentation.to.lactate..formate..ethanol.and.acetate | time_from_admit | time_from_admit | 0.04 | 0.01 | 241 | 229 | 0.000828136 | 0.089231706 |
| P162.PWY..L.glutamate.degradation.V..via.hydroxyglutarate. | read_depth | read_depth | 0.68 | 0.20 | 241 | 98 | 0.000883652 | 0.093652656 |
| DAPLYSINESYN.PWY..L.lysine.biosynthesis.I | covid_severity | Severe | -2.35 | 0.69 | 241 | 198 | 0.000922557 | 0.096198936 |
| PWY.5913..TCA.cycle.VI..obligate.autotrophs. | covid_severity | Severe | -1.96 | 0.59 | 241 | 214 | 0.001068826 | 0.106384584 |
| PWY.7560..methylerythritol.phosphate.pathway.II | time_from_admit | time_from_admit | -0.05 | 0.02 | 241 | 102 | 0.001059311 | 0.106384584 |
| PWY0.1479..tRNA.processing | covid_severity | Severe | -1.93 | 0.58 | 241 | 184 | 0.001069605 | 0.106384584 |
| PWY.5265..peptidoglycan.biosynthesis.II..staphylococci. | covid_severity | Severe | 1.69 | 0.51 | 241 | 187 | 0.001117203 | 0.106455167 |
| PWY.6969..TCA.cycle.V..2.oxoglutarate.ferredoxin.oxidoreductase. | covid_severity | Severe | -1.88 | 0.57 | 241 | 191 | 0.001119714 | 0.106455167 |
| PWY66.389..phytol.degradation | charlson | charlson | 1.26 | 0.37 | 241 | 178 | 0.001116644 | 0.106455167 |
| PWY.5030..L.histidine.degradation.III | time_from_admit | time_from_admit | -0.05 | 0.02 | 241 | 216 | 0.001207111 | 0.112088394 |
| PWY.6185..4.methylcatechol.degradation..ortho.cleavage. | time_from_admit | time_from_admit | 0.04 | 0.01 | 241 | 45 | 0.001213641 | 0.112088394 |
| PWY.622..starch.biosynthesis | covid_severity | Severe | -1.90 | 0.57 | 241 | 73 | 0.001242247 | 0.113114497 |
| CENTFERM.PWY..pyruvate.fermentation.to.butanoate | covid_severity | Severe | -1.73 | 0.53 | 241 | 162 | 0.001329359 | 0.11773026 |
| PWY.6590..superpathway.of.Clostridium.acetobutylicum.acidogenic.fermentation | covid_severity | Severe | -1.72 | 0.52 | 241 | 162 | 0.001321942 | 0.11773026 |
| ARG.POLYAMINE.SYN..superpathway.of.arginine.and.polyamine.biosynthesis | covid_severity | Severe | -2.12 | 0.65 | 241 | 182 | 0.001388061 | 0.121267785 |
| P441.PWY..superpathway.of.N.acetylneuraminate.degradation | covid_severity | Severe | -1.34 | 0.41 | 241 | 208 | 0.001461193 | 0.124297514 |
| PWY.5505..L.glutamate.and.L.glutamine.biosynthesis | time_from_admit | time_from_admit | -0.05 | 0.02 | 241 | 177 | 0.00144588 | 0.124297514 |
| PWY.7211..superpathway.of.pyrimidine.deoxyribonucleotides.de.novo.biosynthesis | race | Black | -1.33 | 0.41 | 241 | 228 | 0.001486413 | 0.124555595 |
| PWY.7400..L.arginine.biosynthesis.IV..archaebacteria. | covid_severity | Severe | -1.35 | 0.42 | 241 | 233 | 0.001502759 | 0.124555595 |
| ARGSYN.PWY..L.arginine.biosynthesis.I..via.L.ornithine. | covid_severity | Severe | -1.19 | 0.37 | 241 | 236 | 0.001747435 | 0.136110415 |
| GLCMANNANAUT.PWY..superpathway.of.N.acetylglucosamine..N.acetylmannosamine.and.N.acetylneuraminate.degradation | covid_severity | Severe | -1.22 | 0.38 | 241 | 228 | 0.001729056 | 0.136110415 |
| POLYAMSYN.PWY..superpathway.of.polyamine.biosynthesis.I | covid_severity | Severe | -2.11 | 0.66 | 241 | 182 | 0.001743481 | 0.136110415 |
| PWY.5136..fatty.acid..beta..oxidation.II..peroxisome. | charlson | charlson | 0.81 | 0.25 | 241 | 209 | 0.001737465 | 0.136110415 |
| PWY.7392..taxadiene.biosynthesis..engineered. | read_depth | read_depth | 0.83 | 0.26 | 241 | 98 | 0.001699588 | 0.136110415 |
| X7ALPHADEHYDROX.PWY..cholate.degradation..bacteria..anaerobic. | read_depth | read_depth | 0.64 | 0.20 | 241 | 69 | 0.001908179 | 0.137609774 |
| GLUDEG.I.PWY..GABA.shunt | covid_severity | Severe | -1.89 | 0.60 | 241 | 190 | 0.001868302 | 0.137609774 |
| NONMEVIPP.PWY..methylerythritol.phosphate.pathway.I | time_from_admit | time_from_admit | -0.06 | 0.02 | 241 | 102 | 0.001915862 | 0.137609774 |
| PROTOCATECHUATE.ORTHO.CLEAVAGE.PWY..protocatechuate.degradation.II..ortho.cleavage.pathway. | time_from_admit | time_from_admit | 0.04 | 0.01 | 241 | 58 | 0.00189055 | 0.137609774 |
| PWY.5677..succinate.fermentation.to.butanoate | read_depth | read_depth | 0.59 | 0.19 | 241 | 127 | 0.001899118 | 0.137609774 |
| PWY.6531..mannitol.cycle | read_depth | read_depth | 0.71 | 0.23 | 241 | 217 | 0.001936967 | 0.137609774 |
| PWY.7209..superpathway.of.pyrimidine.ribonucleosides.degradation | on_abx | TRUE | -1.59 | 0.50 | 241 | 90 | 0.0019051 | 0.137609774 |
| SULFATE.CYS.PWY..superpathway.of.sulfate.assimilation.and.cysteine.biosynthesis | covid_severity | Severe | -1.91 | 0.61 | 241 | 187 | 0.001925013 | 0.137609774 |
| FUCCAT.PWY..fucose.degradation | covid_severity | Severe | -1.84 | 0.59 | 241 | 209 | 0.002102985 | 0.144636168 |
| PWY.5345..superpathway.of.L.methionine.biosynthesis..by.sulfhydrylation. | covid_severity | Severe | -1.78 | 0.57 | 241 | 186 | 0.002095594 | 0.144636168 |
| PWY66.388..fatty.acid..alpha..oxidation.III | age | age | 0.64 | 0.20 | 241 | 43 | 0.002088625 | 0.144636168 |
| PWY.4722..creatinine.degradation.II | read_depth | read_depth | 0.40 | 0.13 | 241 | 81 | 0.002132703 | 0.14513604 |
| NONMEVIPP.PWY..methylerythritol.phosphate.pathway.I | read_depth | read_depth | 0.87 | 0.28 | 241 | 102 | 0.002165594 | 0.145336444 |
| PWY.6270..isoprene.biosynthesis.I | time_from_admit | time_from_admit | -0.06 | 0.02 | 241 | 102 | 0.00220402 | 0.145336444 |
| PWY.6396..superpathway.of.2.3.butanediol.biosynthesis | male | yes | -0.89 | 0.28 | 241 | 53 | 0.00222557 | 0.145336444 |
| PWY66.367..ketogenesis | read_depth | read_depth | 0.53 | 0.17 | 241 | 113 | 0.002186746 | 0.145336444 |
| HEXITOLDEGSUPER.PWY..superpathway.of.hexitol.degradation..bacteria. | covid_severity | Severe | -0.98 | 0.32 | 241 | 229 | 0.002314241 | 0.149615652 |
| FAO.PWY..fatty.acid..beta..oxidation.I | charlson | charlson | 0.79 | 0.25 | 241 | 208 | 0.002569115 | 0.164448766 |
| PWY.6318..L.phenylalanine.degradation.IV..mammalian..via.side.chain. | race | Other | 2.91 | 0.94 | 241 | 173 | 0.002700174 | 0.169481775 |
| PWY.7013..L.1.2.propanediol.degradation | covid_severity | Severe | -1.92 | 0.63 | 241 | 195 | 0.002698989 | 0.169481775 |
| PWY.7332..superpathway.of.UDP.N.acetylglucosamine.derived.O.antigen.building.blocks.biosynthesis | tx_remdesivir | 1 | 1.97 | 0.65 | 241 | 160 | 0.00276303 | 0.171759485 |
| POLYAMINSYN3.PWY..superpathway.of.polyamine.biosynthesis.II | race | Black | 2.06 | 0.67 | 241 | 88 | 0.002926354 | 0.176811953 |
| PWY.5121..superpathway.of.geranylgeranyl.diphosphate.biosynthesis.II..via.MEP. | read_depth | read_depth | 0.77 | 0.25 | 241 | 101 | 0.002904144 | 0.176811953 |
| PWY.7245..superpathway.NAD.NADP...NADH.NADPH.interconversion..yeast. | time_from_admit | time_from_admit | 0.03 | 0.01 | 241 | 25 | 0.002889644 | 0.176811953 |
| PWY.6270..isoprene.biosynthesis.I | read_depth | read_depth | 0.87 | 0.29 | 241 | 102 | 0.003005882 | 0.179935425 |
| PWY.5100..pyruvate.fermentation.to.acetate.and.lactate.II | covid_severity | Severe | -0.99 | 0.33 | 241 | 238 | 0.003160813 | 0.187302168 |
| PWY.7279..aerobic.respiration.II..cytochrome.c...yeast. | charlson | charlson | 1.04 | 0.34 | 241 | 116 | 0.003186889 | 0.187302168 |
| COBALSYN.PWY..adenosylcobalamin.salvage.from.cobinamide.I | covid_severity | Severe | -1.64 | 0.55 | 241 | 216 | 0.003246255 | 0.189072436 |
| TCA..TCA.cycle.I..prokaryotic. | covid_severity | Severe | -1.68 | 0.56 | 241 | 214 | 0.003334304 | 0.192466744 |
| CITRULBIO.PWY..L.citrulline.biosynthesis | tx_remdesivir | 1 | 1.28 | 0.43 | 241 | 233 | 0.003528887 | 0.196795855 |
| PWY.5121..superpathway.of.geranylgeranyl.diphosphate.biosynthesis.II..via.MEP. | time_from_admit | time_from_admit | -0.05 | 0.02 | 241 | 101 | 0.003531063 | 0.196795855 |
| PWY.6182..superpathway.of.salicylate.degradation | time_from_admit | time_from_admit | 0.03 | 0.01 | 241 | 44 | 0.003465901 | 0.196795855 |
| PWY.7560..methylerythritol.phosphate.pathway.II | read_depth | read_depth | 0.73 | 0.25 | 241 | 102 | 0.003509764 | 0.196795855 |
| POLYAMINSYN3.PWY..superpathway.of.polyamine.biosynthesis.II | covid_severity | Severe | -1.61 | 0.54 | 241 | 88 | 0.003577643 | 0.197632206 |
| PWY.6263..superpathway.of.menaquinol.8.biosynthesis.II | tx_remdesivir | 1 | 2.07 | 0.70 | 241 | 140 | 0.003607208 | 0.197632206 |
| CODH.PWY..reductive.acetyl.coenzyme.A.pathway | time_from_admit | time_from_admit | 0.04 | 0.01 | 241 | 90 | 0.003671844 | 0.199482959 |
| P124.PWY..Bifidobacterium.shunt | covid_severity | Severe | -1.89 | 0.64 | 241 | 132 | 0.003752651 | 0.202174061 |
| PWY.7198..pyrimidine.deoxyribonucleotides.de.novo.biosynthesis.IV | read_depth | read_depth | 0.41 | 0.14 | 241 | 235 | 0.003815018 | 0.203835477 |
| PWY.7315..dTDP.N.acetylthomosamine.biosynthesis | covid_severity | Severe | -1.96 | 0.67 | 241 | 181 | 0.003933379 | 0.208436823 |
| GLUTORN.PWY..L.ornithine.biosynthesis | covid_severity | Severe | -1.20 | 0.41 | 241 | 236 | 0.004082988 | 0.21117213 |
| PWY.5028..L.histidine.degradation.II | on_abx | TRUE | 1.36 | 0.47 | 241 | 66 | 0.004068912 | 0.21117213 |
| PWY.7200..superpathway.of.pyrimidine.deoxyribonucleoside.salvage | read_depth | read_depth | -0.44 | 0.15 | 241 | 39 | 0.004070673 | 0.21117213 |
| PWY.6507..4.deoxy.L.threo.hex.4.enopyranuronate.degradation | time_from_admit | time_from_admit | 0.03 | 0.01 | 241 | 226 | 0.004337952 | 0.222578261 |
| PWY.3781..aerobic.respiration.I..cytochrome.c. | charlson | charlson | 1.08 | 0.37 | 241 | 170 | 0.004528117 | 0.227297606 |
| PWY.7392..taxadiene.biosynthesis..engineered. | time_from_admit | time_from_admit | -0.05 | 0.02 | 241 | 98 | 0.004535405 | 0.227297606 |
| SO4ASSIM.PWY..sulfate.reduction.I..assimilatory. | covid_severity | Severe | -1.82 | 0.63 | 241 | 190 | 0.004504012 | 0.227297606 |
| HEXITOLDEGSUPER.PWY..superpathway.of.hexitol.degradation..bacteria. | time_from_admit | time_from_admit | 0.02 | 0.01 | 241 | 229 | 0.004606808 | 0.229100095 |
| PROPFERM.PWY..L.alanine.fermentation.to.propanoate.and.acetate | read_depth | read_depth | 0.22 | 0.08 | 241 | 42 | 0.004864033 | 0.229532634 |
| PRPP.PWY..superpathway.of.histidine..purine..and.pyrimidine.biosynthesis | covid_severity | Severe | -1.05 | 0.37 | 241 | 214 | 0.004772472 | 0.229532634 |
| PWY.5088..L.glutamate.degradation.VIII..to.propanoate. | covid_severity | Severe | -0.99 | 0.35 | 241 | 28 | 0.00483793 | 0.229532634 |
| PWY.5417..catechol.degradation.III..ortho.cleavage.pathway. | time_from_admit | time_from_admit | 0.03 | 0.01 | 241 | 49 | 0.004823022 | 0.229532634 |
| PWY.5431..aromatic.compounds.degradation.via..beta..ketoadipate | time_from_admit | time_from_admit | 0.03 | 0.01 | 241 | 49 | 0.004823022 | 0.229532634 |
| PWY.7268..NAD.NADP.NADH.NADPH.cytosolic.interconversion..yeast. | time_from_admit | time_from_admit | 0.03 | 0.01 | 241 | 42 | 0.004689052 | 0.229532634 |
| PWY3O.19..ubiquinol.6.biosynthesis.from.4.hydroxybenzoate..eukaryotic. | covid_severity | Severe | 1.10 | 0.39 | 241 | 38 | 0.004744333 | 0.229532634 |
| PWY.7210..pyrimidine.deoxyribonucleotides.biosynthesis.from.CTP | charlson | charlson | 0.88 | 0.30 | 241 | 191 | 0.004936598 | 0.231268864 |
| GALACTUROCAT.PWY..D.galacturonate.degradation.I | read_depth | read_depth | 0.47 | 0.17 | 241 | 228 | 0.005019797 | 0.231807034 |
| P125.PWY..superpathway.of..R.R..butanediol.biosynthesis | ethnicity | Hispanic | -1.90 | 0.66 | 241 | 156 | 0.00501123 | 0.231807034 |
| PWY.7456..mannan.degradation | tx_remdesivir | 1 | 1.82 | 0.64 | 241 | 201 | 0.005071322 | 0.232525496 |
| CITRULBIO.PWY..L.citrulline.biosynthesis | ethnicity | Hispanic | -1.21 | 0.42 | 241 | 233 | 0.005141712 | 0.2336575 |
| PWY.6318..L.phenylalanine.degradation.IV..mammalian..via.side.chain. | ethnicity | Hispanic | -1.95 | 0.68 | 241 | 173 | 0.005168294 | 0.2336575 |
| GLUCONEO.PWY..gluconeogenesis.I | covid_severity | Severe | -0.97 | 0.34 | 241 | 233 | 0.005210725 | 0.23393983 |
| PWY.7456..mannan.degradation | time_from_admit | time_from_admit | -0.04 | 0.01 | 241 | 201 | 0.005265382 | 0.234763429 |
| PWY.7013..L.1.2.propanediol.degradation | read_depth | read_depth | 0.72 | 0.26 | 241 | 195 | 0.005433019 | 0.240578527 |
| PWY.6606..guanosine.nucleotides.degradation.II | covid_severity | Severe | -1.22 | 0.43 | 241 | 229 | 0.005472113 | 0.240661306 |
| PWY.6163..chorismate.biosynthesis.from.3.dehydroquinate | time_from_admit | time_from_admit | -0.01 | 0.00 | 241 | 241 | 0.005570249 | 0.243322017 |
| DTDPRHAMSYN.PWY..dTDP.L.rhamnose.biosynthesis.I | charlson | charlson | -0.22 | 0.08 | 241 | 240 | 0.005753383 | 0.247970817 |
| PWY.5005..biotin.biosynthesis.II | read_depth | read_depth | 0.67 | 0.24 | 241 | 153 | 0.005743685 | 0.247970817 |
| Results with FDR-corrected p-value <0.25 shown. | | | | | | | | |

**Supplementary Table 4. Node and network-specific information**

| **Moderate Network** | | | | | | | | | |
| --- | --- | --- | --- | --- | --- | --- | --- | --- | --- |
| **Cluster Color** | **Species** | **Edge Count** | **Degree** | **Betweeness** | **Closeness** | **Eigenvector Centrality** | **Hub Taxon (> 0.8)** | **Hub in Both Networks?** | **Singleton in Both Networks?** |
| Sky blue | *Gordonibacter pamelaeae* | 23 | 0.17 | 0.11 | 1.00 | 0.14 | Yes | Yes |  |
| Sky blue | *Alistipes putredinis* | 22 | 0.16 | 0.08 | 1.00 | 0.15 | Yes |  |  |
| Sky blue | *Odoribacter splanchnicus* | 18 | 0.13 | 0.07 | 0.98 | 0.26 | Yes | Yes |  |
| Sky blue | *Eisenbergiella tayi* | 17 | 0.13 | 0.02 | 0.93 | 0.09 |  |  |  |
| Sky blue | *Bacteroides ovatus* | 14 | 0.10 | 0.01 | 0.94 | 0.27 |  |  |  |
| Sky blue | *Alistipes shahii* | 14 | 0.10 | 0.04 | 0.91 | 0.24 | Yes | Yes |  |
| Sky blue | *Methanobrevibacter smithii* | 13 | 0.10 | 0.06 | 0.84 | 0.09 |  |  |  |
| Sky blue | *Blautia obeum* | 13 | 0.10 | 0.02 | 0.88 | 0.36 |  |  |  |
| Sky blue | *Alistipes finegoldii* | 12 | 0.09 | 0.02 | 0.85 | 0.06 |  |  |  |
| Sky blue | *Bacteroides salyersiae* | 11 | 0.08 | 0.03 | 0.82 | 0.06 |  |  |  |
| Sky blue | *Alistipes indistinctus* | 11 | 0.08 | 0.03 | 0.87 | 0.11 |  |  |  |
| Sky blue | *Butyricimonas synergistica* | 11 | 0.08 | 0.03 | 0.83 | 0.08 |  |  |  |
| Sky blue | *Butyricimonas virosa* | 10 | 0.07 | 0.02 | 0.82 | 0.06 |  |  |  |
| Sky blue | *Bacteroides massiliensis* | 10 | 0.07 | 0.01 | 0.79 | 0.05 | Yes |  |  |
| Sky blue | *Dorea sp CAG 317* | 9 | 0.07 | 0.03 | 0.77 | 0.03 |  |  |  |
| Sky blue | *Monoglobus pectinilyticus* | 9 | 0.07 | 0.01 | 0.76 | 0.04 |  |  |  |
| Sky blue | *Adlercreutzia equolifaciens* | 9 | 0.07 | 0.01 | 0.89 | 0.06 |  |  |  |
| Sky blue | *Eggerthella lenta* | 8 | 0.06 | 0.00 | 0.84 | 0.05 |  |  |  |
| Sky blue | *Asaccharobacter celatus* | 8 | 0.06 | 0.01 | 0.88 | 0.06 |  |  |  |
| Sky blue | *Firmicutes bacterium CAG 145* | 8 | 0.06 | 0.02 | 0.79 | 0.03 |  |  |  |
| Sky blue | *Harryflintia acetispora* | 7 | 0.05 | 0.00 | 0.75 | 0.03 |  |  |  |
| Sky blue | *Clostridium methylpentosum* | 6 | 0.04 | 0.01 | 0.69 | 0.01 |  |  |  |
| Sky blue | *Bacteroides cellulosilyticus* | 6 | 0.04 | 0.00 | 0.73 | 0.02 |  |  |  |
| Sky blue | *Holdemania filiformis* | 5 | 0.04 | 0.00 | 0.74 | 0.04 |  |  |  |
| Sky blue | *Clostridium bolteae* | 5 | 0.04 | 0.02 | 0.72 | 0.05 |  |  |  |
| Sky blue | *Clostridium hylemonae* | 5 | 0.04 | 0.01 | 0.68 | 0.01 |  |  |  |
| Sky blue | *Clostridium lavalense* | 4 | 0.03 | 0.00 | 0.71 | 0.04 |  |  |  |
| Sky blue | *Bacteroides finegoldii* | 4 | 0.03 | 0.00 | 0.67 | 0.01 |  |  |  |
| Sky blue | *Catabacter hongkongensis* | 4 | 0.03 | 0.01 | 0.70 | 0.02 |  |  |  |
| Sky blue | *Anaerofustis stercorihominis* | 3 | 0.02 | 0.00 | 0.66 | 0.01 |  |  |  |
| Sky blue | *Eubacterium siraeum* | 3 | 0.02 | 0.00 | 0.68 | 0.03 |  |  |  |
| Sky blue | *Intestinimonas butyriciproducens* | 2 | 0.01 | 0.00 | 0.61 | 0.01 |  |  |  |
| Sky blue | *Bacteroides dorei* | 2 | 0.01 | 0.00 | 0.67 | 0.03 |  |  |  |
| Sky blue | *Bacteroides fragilis* | 2 | 0.01 | 0.00 | 0.69 | 0.05 |  |  |  |
| Sky blue | *Bacteroides faecis* | 2 | 0.01 | 0.02 | 0.54 | 0.00 |  |  |  |
| Sky blue | *Bacteroides nordii* | 2 | 0.01 | 0.00 | 0.65 | 0.01 |  |  |  |
| Sky blue | *Eubacterium limosum* | 2 | 0.01 | 0.02 | 0.57 | 0.00 |  |  |  |
| Sky blue | *Streptococcus thermophilus* | 1 | 0.01 | 0.00 | 0.54 | 0.00 |  |  |  |
| Sky blue | *Dielma fastidiosa* | 1 | 0.01 | 0.00 | 0.56 | 0.00 |  |  |  |
| Sky blue | *Eubacterium callanderi* | 1 | 0.01 | 0.00 | 0.43 | 0.00 |  |  |  |
| Sky blue | *Bacteroides faecis CAG 32* | 1 | 0.01 | 0.00 | 0.43 | 0.00 |  |  |  |
| Sky blue | *Bacteroides caccae* | 1 | 0.01 | 0.00 | 0.56 | 0.00 |  |  |  |
| Sky blue | *Clostridium bolteae CAG 59* | 1 | 0.01 | 0.00 | 0.53 | 0.00 |  |  |  |
| Purple | *Bacteroides uniformis* | 25 | 0.18 | 0.15 | 1.07 | 0.32 | Yes |  |  |
| Purple | *Eisenbergiella massiliensis* | 20 | 0.15 | 0.06 | 0.94 | 0.09 |  |  |  |
| Purple | *Blautia sp CAG 257* | 17 | 0.13 | 0.01 | 0.83 | 0.14 |  |  |  |
| Purple | *Sellimonas intestinalis* | 16 | 0.12 | 0.02 | 0.84 | 0.07 |  |  |  |
| Purple | *Blautia producta* | 15 | 0.11 | 0.03 | 0.82 | 0.11 |  |  |  |
| Purple | *Streptococcus salivarius* | 12 | 0.09 | 0.06 | 0.89 | 0.19 |  |  |  |
| Purple | *Anaerotruncus colihominis* | 11 | 0.08 | 0.05 | 0.83 | 0.04 |  |  |  |
| Purple | *Streptococcus gordonii* | 11 | 0.08 | 0.02 | 0.82 | 0.06 |  |  |  |
| Purple | *Parabacteroides distasonis* | 11 | 0.08 | 0.04 | 0.87 | 0.07 |  |  |  |
| Purple | *Bacteroides thetaiotaomicron* | 11 | 0.08 | 0.01 | 0.89 | 0.24 |  |  |  |
| Purple | *Clostridium scindens* | 11 | 0.08 | 0.04 | 0.78 | 0.07 |  |  |  |
| Purple | *Blautia hydrogenotrophica* | 10 | 0.07 | 0.00 | 0.78 | 0.04 |  |  |  |
| Purple | *Bacteroides stercoris* | 10 | 0.07 | 0.02 | 0.83 | 0.05 |  |  |  |
| Purple | *Lawsonibacter asaccharolyticus* | 9 | 0.07 | 0.00 | 0.84 | 0.07 |  |  |  |
| Purple | *Clostridium symbiosum* | 9 | 0.07 | 0.00 | 0.81 | 0.05 |  |  |  |
| Purple | *Anaerostipes caccae* | 9 | 0.07 | 0.00 | 0.77 | 0.06 |  |  |  |
| Purple | *Clostridium innocuum* | 9 | 0.07 | 0.01 | 0.74 | 0.05 |  |  |  |
| Purple | *Parabacteroides merdae* | 8 | 0.06 | 0.01 | 0.84 | 0.10 |  |  |  |
| Purple | *Ruminococcus gnavus* | 8 | 0.06 | 0.01 | 0.72 | 0.03 |  |  |  |
| Purple | *Streptococcus parasanguinis* | 8 | 0.06 | 0.01 | 0.72 | 0.03 |  |  |  |
| Purple | *Flavonifractor plautii* | 7 | 0.05 | 0.00 | 0.81 | 0.08 |  |  |  |
| Purple | *Ruthenibacterium lactatiformans* | 7 | 0.05 | 0.02 | 0.78 | 0.04 |  |  |  |
| Purple | *Clostridium spiroforme* | 6 | 0.04 | 0.00 | 0.73 | 0.02 |  |  |  |
| Purple | *Streptococcus vestibularis* | 6 | 0.04 | 0.01 | 0.71 | 0.02 |  |  |  |
| Purple | *Actinomyces odontolyticus* | 6 | 0.04 | 0.00 | 0.72 | 0.06 |  |  |  |
| Purple | *Bacteroides vulgatus* | 6 | 0.04 | 0.01 | 0.78 | 0.06 |  |  |  |
| Purple | *Actinomyces oris* | 5 | 0.04 | 0.00 | 0.66 | 0.02 |  |  |  |
| Purple | *Rothia mucilaginosa* | 5 | 0.04 | 0.00 | 0.67 | 0.02 |  |  |  |
| Purple | *Bacteroides xylanisolvens* | 4 | 0.03 | 0.01 | 0.69 | 0.03 |  |  |  |
| Purple | *Clostridium leptum* | 3 | 0.02 | 0.02 | 0.67 | 0.02 |  |  |  |
| Purple | *Clostridium clostridioforme* | 3 | 0.02 | 0.00 | 0.61 | 0.00 |  |  |  |
| Purple | *Erysipelatoclostridium ramosum* | 3 | 0.02 | 0.00 | 0.68 | 0.01 |  |  |  |
| Purple | *Anaerotignum lactatifermentans* | 3 | 0.02 | 0.00 | 0.70 | 0.02 |  |  |  |
| Purple | *Bilophila wadsworthia* | 3 | 0.02 | 0.00 | 0.70 | 0.02 |  |  |  |
| Purple | *Clostridium citroniae* | 2 | 0.01 | 0.00 | 0.66 | 0.06 |  |  |  |
| Purple | *Ruminococcaceae bacterium D16* | 2 | 0.01 | 0.02 | 0.57 | 0.00 |  |  |  |
| Purple | *Clostridium asparagiforme* | 1 | 0.01 | 0.00 | 0.43 | 0.00 |  |  |  |
| Purple | *Klebsiella pneumoniae* | 1 | 0.01 | 0.00 | 0.42 | 0.00 |  |  |  |
| Gold | *Blautia coccoides* | 5 | 0.04 | 0.03 | 0.60 | 0.01 |  |  |  |
| Gold | *Enterococcus faecium* | 4 | 0.03 | 0.01 | 0.61 | 0.03 |  |  |  |
| Gold | *Lactobacillus rhamnosus* | 4 | 0.03 | 0.01 | 0.54 | 0.00 |  |  |  |
| Gold | *Enterococcus faecalis* | 2 | 0.01 | 0.00 | 0.47 | 0.00 |  |  |  |
| Gold | *Barnesiella intestinihominis* | 1 | 0.01 | 0.00 | 0.39 | 0.00 |  |  |  |
| Pink | *Lactobacillus fermentum* | 9 | 0.07 | 0.03 | 0.63 | 0.06 |  |  |  |
| Pink | *Veillonella parvula* | 7 | 0.05 | 0.02 | 0.62 | 0.00 |  |  |  |
| Pink | *Streptococcus anginosus group* | 6 | 0.04 | 0.02 | 0.70 | 0.01 |  |  |  |
| Pink | *Parabacteroides goldsteinii* | 5 | 0.04 | 0.01 | 0.68 | 0.01 |  |  |  |
| Pink | *Lactobacillus paragasseri* | 5 | 0.04 | 0.00 | 0.56 | 0.01 |  |  |  |
| Pink | *Candida albicans* | 5 | 0.04 | 0.01 | 0.63 | 0.03 |  |  |  |
| Pink | *Bifidobacterium longum* | 4 | 0.03 | 0.01 | 0.68 | 0.05 |  |  |  |
| Pink | *Lactobacillus gasseri* | 4 | 0.03 | 0.00 | 0.53 | 0.00 |  |  |  |
| Pink | *Bifidobacterium breve* | 4 | 0.03 | 0.00 | 0.53 | 0.00 |  |  |  |
| Pink | *Phascolarctobacterium faecium* | 3 | 0.02 | 0.00 | 0.66 | 0.01 |  |  |  |
| Green | *Faecalibacterium prausnitzii* | 28 | 0.21 | 0.05 | 1.04 | 0.96 | Yes |  |  |
| Green | *Dorea formicigenerans* | 27 | 0.20 | 0.03 | 1.04 | 1.00 | Yes |  |  |
| Green | *Fusicatenibacter saccharivorans* | 25 | 0.18 | 0.02 | 0.97 | 0.91 |  |  |  |
| Green | *Anaerostipes hadrus* | 23 | 0.17 | 0.03 | 1.00 | 0.85 | Yes |  |  |
| Green | *Gemmiger formicilis* | 22 | 0.16 | 0.00 | 0.94 | 0.89 |  |  |  |
| Green | *Ruminococcus torques* | 21 | 0.15 | 0.02 | 0.91 | 0.74 |  |  |  |
| Green | *Eubacterium hallii* | 21 | 0.15 | 0.05 | 1.00 | 0.78 | Yes | Yes |  |
| Green | *Coprococcus catus* | 21 | 0.15 | 0.01 | 0.91 | 0.83 |  |  |  |
| Green | *Eubacterium rectale* | 21 | 0.15 | 0.04 | 0.97 | 0.76 | Yes |  |  |
| Green | *Blautia wexlerae* | 20 | 0.15 | 0.05 | 0.95 | 0.67 | Yes | Yes |  |
| Green | *Roseburia hominis* | 20 | 0.15 | 0.01 | 0.97 | 0.75 |  |  |  |
| Green | *Roseburia inulinivorans* | 20 | 0.15 | 0.01 | 0.92 | 0.81 |  |  |  |
| Green | *Coprococcus comes* | 20 | 0.15 | 0.01 | 0.89 | 0.80 |  |  |  |
| Green | *Oscillibacter sp 57 20* | 19 | 0.14 | 0.01 | 0.86 | 0.66 |  |  |  |
| Green | *Collinsella aerofaciens* | 17 | 0.13 | 0.02 | 0.91 | 0.62 |  |  |  |
| Green | *Oscillibacter sp CAG 241* | 16 | 0.12 | 0.01 | 0.84 | 0.47 |  |  |  |
| Green | *Dorea longicatena* | 16 | 0.12 | 0.00 | 0.89 | 0.68 |  |  |  |
| Green | *Agathobaculum butyriciproducens* | 15 | 0.11 | 0.00 | 0.84 | 0.52 |  |  |  |
| Green | *Eubacterium ramulus* | 15 | 0.11 | 0.00 | 0.85 | 0.67 |  |  |  |
| Green | *Eubacterium eligens* | 13 | 0.10 | 0.01 | 0.86 | 0.49 |  |  |  |
| Green | *Prevotella copri* | 13 | 0.10 | 0.00 | 0.77 | 0.39 |  |  |  |
| Green | *Clostridium sp CAG 58* | 12 | 0.09 | 0.00 | 0.86 | 0.37 |  |  |  |
| Green | *Roseburia intestinalis* | 10 | 0.07 | 0.00 | 0.78 | 0.41 |  |  |  |
| Green | *Collinsella stercoris* | 8 | 0.06 | 0.03 | 0.82 | 0.11 |  |  |  |
| Green | *Roseburia faecis* | 7 | 0.05 | 0.01 | 0.74 | 0.24 |  |  |  |
| Green | *Bifidobacterium adolescentis* | 6 | 0.04 | 0.00 | 0.74 | 0.14 |  |  |  |
| Green | *Holdemanella biformis* | 6 | 0.04 | 0.00 | 0.68 | 0.23 |  |  |  |
| Green | *Akkermansia muciniphila* | 3 | 0.02 | 0.00 | 0.71 | 0.08 |  |  |  |
| Green | *Ruminococcus bromii* | 3 | 0.02 | 0.00 | 0.68 | 0.12 |  |  |  |
| Green | *Firmicutes bacterium CAG 83* | 3 | 0.02 | 0.00 | 0.67 | 0.14 |  |  |  |
| Violet red | *Parasutterella excrementihominis* | 3 | 0.02 | 0.02 | 0.63 | 0.00 |  |  |  |
| Violet red | *Turicimonas muris* | 3 | 0.02 | 0.00 | 0.56 | 0.00 |  |  |  |
| Violet red | *Proteobacteria bacterium CAG 139* | 2 | 0.01 | 0.00 | 0.56 | 0.00 |  |  |  |
| Grey | *Pseudomonas aeruginosa group* | NA | 0.00 | 0.00 | 0.00 | 0.00 |  |  | Yes |
| Grey | *Hungatella hathewayi* | NA | 0.00 | 0.00 | 0.00 | 0.00 |  |  |  |
| Grey | *Escherichia coli* | NA | 0.00 | 0.00 | 0.00 | 0.00 |  |  |  |
| Grey | *Paraprevotella xylaniphila* | NA | 0.00 | 0.00 | 0.00 | 0.00 |  |  |  |
| Grey | *Coprobacillus cateniformis* | NA | 0.00 | 0.00 | 0.00 | 0.00 |  |  | Yes |
| Grey | *Faecalicatena orotica* | NA | 0.00 | 0.00 | 0.00 | 0.00 |  |  |  |
| Grey | *Acidaminococcus intestini* | NA | 0.00 | 0.00 | 0.00 | 0.00 |  |  | Yes |
| Grey | *Christensenella minuta* | NA | 0.00 | 0.00 | 0.00 | 0.00 |  |  |  |
| **Severe Network** | | | | | | | | | |
| **Cluster Color** | **Species** | **Edge Count** | **Degree** | **Betweeness** | **Closeness** | **Eigenvector Centrality** | **Hub Taxon (> 0.8)** | **Hub in Both Networks?** | **Singleton in Both Networks?** |
| Brick red | *Streptococcus salivarius* | 21 | 0.15 | 0.04 | 1.00 | 0.64 | Yes |  |  |
| Brick red | *Streptococcus parasanguinis* | 16 | 0.12 | 0.03 | 0.96 | 0.40 |  |  |  |
| Brick red | *Anaerotruncus colihominis* | 13 | 0.10 | 0.03 | 0.91 | 0.35 |  |  |  |
| Brick red | *Actinomyces oris* | 12 | 0.09 | 0.03 | 0.84 | 0.22 |  |  |  |
| Brick red | *Streptococcus vestibularis* | 11 | 0.08 | 0.00 | 0.86 | 0.32 |  |  |  |
| Brick red | *Eisenbergiella massiliensis* | 11 | 0.08 | 0.05 | 0.89 | 0.18 |  |  |  |
| Brick red | *Dorea sp CAG 317* | 9 | 0.07 | 0.01 | 0.83 | 0.21 |  |  |  |
| Brick red | *Actinomyces odontolyticus* | 9 | 0.07 | 0.00 | 0.83 | 0.25 |  |  |  |
| Brick red | *Streptococcus gordonii* | 7 | 0.05 | 0.00 | 0.78 | 0.19 |  |  |  |
| Brick red | *Anaerofustis stercorihominis* | 6 | 0.04 | 0.02 | 0.75 | 0.05 |  |  |  |
| Brick red | *Streptococcus anginosus group* | 5 | 0.04 | 0.00 | 0.72 | 0.06 |  |  |  |
| Brick red | *Oscillibacter sp 57 20* | 5 | 0.04 | 0.00 | 0.73 | 0.16 |  |  |  |
| Brick red | *Ruminococcaceae bacterium D16* | 5 | 0.04 | 0.00 | 0.72 | 0.11 |  |  |  |
| Brick red | *Monoglobus pectinilyticus* | 4 | 0.03 | 0.00 | 0.67 | 0.07 |  |  |  |
| Brick red | *Clostridium asparagiforme* | 3 | 0.02 | 0.04 | 0.64 | 0.01 |  |  |  |
| Brick red | *Blautia sp CAG 257* | 2 | 0.01 | 0.00 | 0.59 | 0.01 |  |  |  |
| Brick red | *Bacteroides faecis* | 2 | 0.01 | 0.02 | 0.49 | 0.00 |  |  |  |
| Brick red | *Rothia mucilaginosa* | 2 | 0.01 | 0.00 | 0.63 | 0.03 |  |  |  |
| Brick red | *Bacteroides faecis CAG 32* | 1 | 0.01 | 0.00 | 0.41 | 0.00 |  |  |  |
| Green | *Collinsella aerofaciens* | 29 | 0.21 | 0.07 | 1.10 | 0.95 | Yes |  |  |
| Green | *Roseburia inulinivorans* | 29 | 0.21 | 0.05 | 1.07 | 1.00 | Yes |  |  |
| Green | *Eubacterium hallii* | 26 | 0.19 | 0.10 | 1.08 | 0.83 | Yes | Yes |  |
| Green | *Blautia obeum* | 25 | 0.18 | 0.08 | 1.09 | 0.90 | Yes |  |  |
| Green | *Dorea longicatena* | 23 | 0.17 | 0.02 | 0.99 | 0.83 |  |  |  |
| Green | *Collinsella stercoris* | 22 | 0.16 | 0.03 | 1.01 | 0.78 |  |  |  |
| Green | *Ruminococcus torques* | 22 | 0.16 | 0.03 | 1.02 | 0.89 |  |  |  |
| Green | *Anaerostipes hadrus* | 22 | 0.16 | 0.01 | 1.00 | 0.85 |  |  |  |
| Green | *Coprococcus catus* | 22 | 0.16 | 0.01 | 0.98 | 0.85 |  |  |  |
| Green | *Blautia wexlerae* | 20 | 0.15 | 0.04 | 0.97 | 0.70 | Yes | Yes |  |
| Green | *Dorea formicigenerans* | 20 | 0.15 | 0.02 | 0.99 | 0.83 |  |  |  |
| Green | *Ruminococcus bromii* | 19 | 0.14 | 0.02 | 0.95 | 0.70 |  |  |  |
| Green | *Alistipes shahii* | 19 | 0.14 | 0.07 | 0.98 | 0.55 | Yes | Yes |  |
| Green | *Eubacterium eligens* | 18 | 0.13 | 0.01 | 0.94 | 0.63 |  |  |  |
| Green | *Intestinimonas butyriciproducens* | 16 | 0.12 | 0.02 | 0.94 | 0.44 |  |  |  |
| Green | *Faecalibacterium prausnitzii* | 16 | 0.12 | 0.03 | 0.92 | 0.55 |  |  |  |
| Green | *Eubacterium rectale* | 16 | 0.12 | 0.00 | 0.89 | 0.57 |  |  |  |
| Green | *Coprococcus comes* | 15 | 0.11 | 0.01 | 0.90 | 0.51 |  |  |  |
| Green | *Bifidobacterium longum* | 14 | 0.10 | 0.02 | 0.92 | 0.40 |  |  |  |
| Green | *Fusicatenibacter saccharivorans* | 13 | 0.10 | 0.00 | 0.89 | 0.63 |  |  |  |
| Green | *Gemmiger formicilis* | 12 | 0.09 | 0.02 | 0.85 | 0.42 |  |  |  |
| Green | *Eubacterium ramulus* | 12 | 0.09 | 0.00 | 0.83 | 0.43 |  |  |  |
| Green | *Roseburia hominis* | 11 | 0.08 | 0.00 | 0.84 | 0.37 |  |  |  |
| Green | *Agathobaculum butyriciproducens* | 7 | 0.05 | 0.00 | 0.79 | 0.31 |  |  |  |
| Green | *Adlercreutzia equolifaciens* | 7 | 0.05 | 0.00 | 0.82 | 0.23 |  |  |  |
| Green | *Asaccharobacter celatus* | 7 | 0.05 | 0.00 | 0.82 | 0.23 |  |  |  |
| Green | *Bifidobacterium adolescentis* | 7 | 0.05 | 0.00 | 0.78 | 0.27 |  |  |  |
| Green | *Bilophila wadsworthia* | 6 | 0.04 | 0.01 | 0.78 | 0.22 |  |  |  |
| Green | *Roseburia faecis* | 6 | 0.04 | 0.00 | 0.74 | 0.18 |  |  |  |
| Green | *Holdemanella biformis* | 6 | 0.04 | 0.00 | 0.77 | 0.21 |  |  |  |
| Green | *Roseburia intestinalis* | 5 | 0.04 | 0.00 | 0.71 | 0.10 |  |  |  |
| Green | *Escherichia coli* | 2 | 0.01 | 0.00 | 0.69 | 0.09 |  |  |  |
| Green | *Prevotella copri* | 1 | 0.01 | 0.00 | 0.57 | 0.02 |  |  |  |
| Light blue | *Odoribacter splanchnicus* | 15 | 0.11 | 0.08 | 0.92 | 0.23 | Yes | Yes |  |
| Light blue | *Alistipes indistinctus* | 14 | 0.10 | 0.09 | 0.90 | 0.14 |  |  |  |
| Light blue | *Oscillibacter sp CAG 241* | 10 | 0.07 | 0.03 | 0.85 | 0.22 |  |  |  |
| Light blue | *Eubacterium siraeum* | 9 | 0.07 | 0.01 | 0.84 | 0.19 |  |  |  |
| Light blue | *Parabacteroides merdae* | 7 | 0.05 | 0.02 | 0.78 | 0.10 |  |  |  |
| Light blue | *Butyricimonas virosa* | 7 | 0.05 | 0.04 | 0.78 | 0.06 |  |  |  |
| Light blue | *Alistipes putredinis* | 7 | 0.05 | 0.01 | 0.70 | 0.03 |  |  |  |
| Light blue | *Barnesiella intestinihominis* | 7 | 0.05 | 0.03 | 0.75 | 0.03 |  |  |  |
| Light blue | *Alistipes finegoldii* | 6 | 0.04 | 0.03 | 0.74 | 0.05 |  |  |  |
| Light blue | *Bacteroides caccae* | 6 | 0.04 | 0.02 | 0.70 | 0.02 |  |  |  |
| Light blue | *Bacteroides dorei* | 4 | 0.03 | 0.00 | 0.65 | 0.01 |  |  |  |
| Light blue | *Methanobrevibacter smithii* | 4 | 0.03 | 0.01 | 0.68 | 0.02 |  |  |  |
| Light blue | *Bacteroides vulgatus* | 4 | 0.03 | 0.00 | 0.61 | 0.01 |  |  |  |
| Light blue | *Bacteroides xylanisolvens* | 4 | 0.03 | 0.00 | 0.66 | 0.02 |  |  |  |
| Light blue | *Bacteroides nordii* | 4 | 0.03 | 0.02 | 0.68 | 0.02 |  |  |  |
| Light blue | *Parabacteroides distasonis* | 4 | 0.03 | 0.00 | 0.63 | 0.02 |  |  |  |
| Light blue | *Eubacterium limosum* | 4 | 0.03 | 0.02 | 0.68 | 0.01 |  |  |  |
| Light blue | *Butyricimonas synergistica* | 4 | 0.03 | 0.04 | 0.65 | 0.01 |  |  |  |
| Light blue | *Phascolarctobacterium faecium* | 3 | 0.02 | 0.02 | 0.56 | 0.00 |  |  |  |
| Light blue | *Parabacteroides goldsteinii* | 3 | 0.02 | 0.00 | 0.69 | 0.04 |  |  |  |
| Light blue | *Bacteroides finegoldii* | 3 | 0.02 | 0.02 | 0.51 | 0.00 |  |  |  |
| Light blue | *Christensenella minuta* | 3 | 0.02 | 0.00 | 0.63 | 0.01 |  |  |  |
| Light blue | *Clostridium sp CAG 58* | 3 | 0.02 | 0.01 | 0.69 | 0.08 |  |  |  |
| Light blue | *Bacteroides massiliensis* | 2 | 0.01 | 0.01 | 0.55 | 0.00 |  |  |  |
| Light blue | *Holdemania filiformis* | 1 | 0.01 | 0.00 | 0.43 | 0.00 |  |  |  |
| Light blue | *Bacteroides stercoris* | 1 | 0.01 | 0.00 | 0.50 | 0.00 |  |  |  |
| Light blue | *Paraprevotella xylaniphila* | 1 | 0.01 | 0.00 | 0.40 | 0.00 |  |  |  |
| Light blue | *Veillonella parvula* | 1 | 0.01 | 0.00 | 0.49 | 0.00 |  |  |  |
| Steel blue | *Firmicutes bacterium CAG 83* | 8 | 0.06 | 0.03 | 0.84 | 0.16 |  |  |  |
| Steel blue | *Faecalicatena orotica* | 3 | 0.02 | 0.00 | 0.62 | 0.01 |  |  |  |
| Steel blue | *Clostridium hylemonae* | 2 | 0.01 | 0.00 | 0.58 | 0.01 |  |  |  |
| Purple | *Gordonibacter pamelaeae* | 21 | 0.15 | 0.08 | 1.01 | 0.32 | Yes | Yes |  |
| Purple | *Flavonifractor plautii* | 13 | 0.10 | 0.04 | 0.86 | 0.08 |  |  |  |
| Purple | *Clostridium leptum* | 12 | 0.09 | 0.03 | 0.92 | 0.18 |  |  |  |
| Purple | *Ruminococcus gnavus* | 12 | 0.09 | 0.03 | 0.88 | 0.18 |  |  |  |
| Purple | *Clostridium scindens* | 12 | 0.09 | 0.04 | 0.85 | 0.07 |  |  |  |
| Purple | *Eggerthella lenta* | 11 | 0.08 | 0.03 | 0.92 | 0.13 |  |  |  |
| Purple | *Clostridium lavalense* | 11 | 0.08 | 0.06 | 0.85 | 0.06 |  |  |  |
| Purple | *Eisenbergiella tayi* | 10 | 0.07 | 0.01 | 0.85 | 0.17 |  |  |  |
| Purple | *Erysipelatoclostridium ramosum* | 9 | 0.07 | 0.01 | 0.77 | 0.04 |  |  |  |
| Purple | *Clostridium bolteae* | 9 | 0.07 | 0.04 | 0.80 | 0.03 |  |  |  |
| Purple | *Ruthenibacterium lactatiformans* | 8 | 0.06 | 0.00 | 0.78 | 0.06 |  |  |  |
| Purple | *Lawsonibacter asaccharolyticus* | 7 | 0.05 | 0.01 | 0.79 | 0.07 |  |  |  |
| Purple | *Blautia producta* | 7 | 0.05 | 0.00 | 0.73 | 0.04 |  |  |  |
| Purple | *Bacteroides uniformis* | 7 | 0.05 | 0.02 | 0.74 | 0.02 |  |  |  |
| Purple | *Hungatella hathewayi* | 6 | 0.04 | 0.01 | 0.74 | 0.04 |  |  |  |
| Purple | *Blautia coccoides* | 6 | 0.04 | 0.01 | 0.73 | 0.02 |  |  |  |
| Purple | *Catabacter hongkongensis* | 6 | 0.04 | 0.01 | 0.70 | 0.01 |  |  |  |
| Purple | *Clostridium citroniae* | 5 | 0.04 | 0.00 | 0.67 | 0.01 |  |  |  |
| Purple | *Parasutterella excrementihominis* | 5 | 0.04 | 0.01 | 0.72 | 0.01 |  |  |  |
| Purple | *Clostridium innocuum* | 5 | 0.04 | 0.00 | 0.71 | 0.04 |  |  |  |
| Purple | *Clostridium spiroforme* | 4 | 0.03 | 0.01 | 0.70 | 0.05 |  |  |  |
| Purple | *Streptococcus thermophilus* | 4 | 0.03 | 0.01 | 0.68 | 0.02 |  |  |  |
| Purple | *Clostridium symbiosum* | 4 | 0.03 | 0.00 | 0.70 | 0.02 |  |  |  |
| Purple | *Turicimonas muris* | 4 | 0.03 | 0.00 | 0.69 | 0.01 |  |  |  |
| Purple | *Blautia hydrogenotrophica* | 3 | 0.02 | 0.00 | 0.68 | 0.04 |  |  |  |
| Purple | *Bacteroides ovatus* | 3 | 0.02 | 0.00 | 0.72 | 0.06 |  |  |  |
| Purple | *Firmicutes bacterium CAG 145* | 3 | 0.02 | 0.00 | 0.66 | 0.02 |  |  |  |
| Purple | *Proteobacteria bacterium CAG 139* | 3 | 0.02 | 0.00 | 0.67 | 0.01 |  |  |  |
| Purple | *Clostridium bolteae CAG 59* | 3 | 0.02 | 0.00 | 0.62 | 0.00 |  |  |  |
| Purple | *Sellimonas intestinalis* | 2 | 0.01 | 0.00 | 0.65 | 0.01 |  |  |  |
| Pink | *Lactobacillus paragasseri* | 3 | 0.02 | 0.02 | 0.67 | 0.05 |  |  |  |
| Pink | *Lactobacillus fermentum* | 3 | 0.02 | 0.00 | 0.67 | 0.05 |  |  |  |
| Pink | *Lactobacillus gasseri* | 2 | 0.01 | 0.00 | 0.53 | 0.01 |  |  |  |
| Light grey | *Enterococcus faecalis* | NA | 0.00 | 0.00 | 0.00 | 0.00 |  |  |  |
| Light grey | *Akkermansia muciniphila* | NA | 0.00 | 0.00 | 0.00 | 0.00 |  |  |  |
| Light grey | *Dielma fastidiosa* | NA | 0.00 | 0.00 | 0.00 | 0.00 |  |  |  |
| Light grey | *Enterococcus faecium* | NA | 0.00 | 0.00 | 0.00 | 0.00 |  |  |  |
| Light grey | *Harryflintia acetispora* | NA | 0.00 | 0.00 | 0.00 | 0.00 |  |  |  |
| Light grey | *Pseudomonas aeruginosa group* | NA | 0.00 | 0.00 | 0.00 | 0.00 |  |  | Yes |
| Light grey | *Clostridium clostridioforme* | NA | 0.00 | 0.00 | 0.00 | 0.00 |  |  |  |
| Light grey | *Bacteroides fragilis* | NA | 0.00 | 0.00 | 0.00 | 0.00 |  |  |  |
| Light grey | *Bacteroides salyersiae* | NA | 0.00 | 0.00 | 0.00 | 0.00 |  |  |  |
| Light grey | *Lactobacillus rhamnosus* | NA | 0.00 | 0.00 | 0.00 | 0.00 |  |  |  |
| Light grey | *Anaerotignum lactatifermentans* | NA | 0.00 | 0.00 | 0.00 | 0.00 |  |  |  |
| Light grey | *Bacteroides thetaiotaomicron* | NA | 0.00 | 0.00 | 0.00 | 0.00 |  |  |  |
| Light grey | *Eubacterium callanderi* | NA | 0.00 | 0.00 | 0.00 | 0.00 |  |  |  |
| Light grey | *Klebsiella pneumoniae* | NA | 0.00 | 0.00 | 0.00 | 0.00 |  |  |  |
| Light grey | *Coprobacillus cateniformis* | NA | 0.00 | 0.00 | 0.00 | 0.00 |  |  | Yes |
| Light grey | *Clostridium methylpentosum* | NA | 0.00 | 0.00 | 0.00 | 0.00 |  |  |  |
| Light grey | *Bifidobacterium breve* | NA | 0.00 | 0.00 | 0.00 | 0.00 |  |  |  |
| Light grey | *Anaerostipes caccae* | NA | 0.00 | 0.00 | 0.00 | 0.00 |  |  |  |
| Light grey | *Bacteroides cellulosilyticus* | NA | 0.00 | 0.00 | 0.00 | 0.00 |  |  |  |
| Light grey | *Acidaminococcus intestini* | NA | 0.00 | 0.00 | 0.00 | 0.00 |  |  | Yes |
| Light grey | *Candida albicans* | NA | 0.00 | 0.00 | 0.00 | 0.00 |  |  |  |

**Supplementary Table 5. Multivariable linear modeling results (predicted stool metabolites)**

| **feature** | **metadata** | **value** | **coef** | **stderr** | **N** | **N.not.0** | **pval** | **qval** |
| --- | --- | --- | --- | --- | --- | --- | --- | --- |
| adrenic.acid | covid_severity | Severe | 0.78 | 0.11 | 241 | 241 | 2.01E-10 | 2.57E-07 |
| eicosatrienoic.acid | covid_severity | Severe | 0.70 | 0.10 | 241 | 241 | 6.81E-10 | 4.36E-07 |
| docosapentaenoic.acid | covid_severity | Severe | 0.38 | 0.06 | 241 | 241 | 6.32E-09 | 2.70E-06 |
| arachidonic.acid | covid_severity | Severe | 0.37 | 0.06 | 241 | 241 | 4.97E-08 | 1.16E-05 |
| C18.0.SM | covid_severity | Severe | 0.29 | 0.05 | 241 | 241 | 5.73E-08 | 1.16E-05 |
| thymine | covid_severity | Severe | -0.21 | 0.04 | 241 | 241 | 6.12E-08 | 1.16E-05 |
| bilirubin | covid_severity | Severe | 0.26 | 0.05 | 241 | 241 | 6.32E-08 | 1.16E-05 |
| palmitoyl.glycerol | covid_severity | Severe | 0.24 | 0.04 | 241 | 241 | 7.46E-08 | 1.19E-05 |
| C18.1.CE | covid_severity | Severe | 0.31 | 0.05 | 241 | 241 | 8.63E-08 | 1.22E-05 |
| docosapentaenoate | covid_severity | Severe | 0.29 | 0.05 | 241 | 241 | 9.54E-08 | 1.22E-05 |
| docosahexaenoic.acid | covid_severity | Severe | 0.21 | 0.04 | 241 | 241 | 1.27E-07 | 1.48E-05 |
| dimethyllysine | covid_severity | Severe | 0.26 | 0.05 | 241 | 241 | 2.83E-07 | 3.02E-05 |
| undecanedionate | covid_severity | Severe | -0.49 | 0.09 | 241 | 241 | 3.28E-07 | 3.23E-05 |
| C16.0.LPC | covid_severity | Severe | 0.53 | 0.10 | 241 | 241 | 3.91E-07 | 3.33E-05 |
| erythronic.acid | covid_severity | Severe | 0.21 | 0.04 | 241 | 241 | 3.84E-07 | 3.33E-05 |
| malonate | covid_severity | Severe | 0.24 | 0.05 | 241 | 241 | 5.59E-07 | 4.21E-05 |
| ADMA | covid_severity | Severe | 0.53 | 0.10 | 241 | 241 | 5.30E-07 | 4.21E-05 |
| C16.0.ceramide..d18.1. | covid_severity | Severe | 0.26 | 0.05 | 241 | 241 | 1.16E-06 | 7.79E-05 |
| pantothenate | covid_severity | Severe | 0.14 | 0.03 | 241 | 241 | 1.15E-06 | 7.79E-05 |
| C18.0e.MAG | covid_severity | Severe | 0.22 | 0.04 | 241 | 241 | 1.29E-06 | 8.27E-05 |
| pyridoxamine | covid_severity | Severe | -0.30 | 0.06 | 241 | 241 | 1.47E-06 | 8.97E-05 |
| diacetylspermine | covid_severity | Severe | 0.38 | 0.08 | 241 | 241 | 1.95E-06 | 0.000113243 |
| imidazole.propionate | covid_severity | Severe | 0.46 | 0.09 | 241 | 241 | 2.45E-06 | 0.000136517 |
| xanthine | read_depth | read_depth | -0.07 | 0.01 | 241 | 241 | 2.91E-06 | 0.000155296 |
| X2.hydroxyphenethylamine | covid_severity | Severe | 0.26 | 0.06 | 241 | 241 | 3.86E-06 | 0.00019768 |
| azelate | covid_severity | Severe | -0.21 | 0.04 | 241 | 241 | 4.14E-06 | 0.000204031 |
| C2.carnitine | covid_severity | Severe | 0.98 | 0.21 | 241 | 241 | 5.05E-06 | 0.000239389 |
| N.acetylputrescine | covid_severity | Severe | 0.81 | 0.17 | 241 | 241 | 6.67E-06 | 0.000301382 |
| creatine | covid_severity | Severe | 0.61 | 0.13 | 241 | 241 | 6.83E-06 | 0.000301382 |
| cholate | covid_severity | Severe | 1.00 | 0.22 | 241 | 241 | 8.32E-06 | 0.000354993 |
| caproic.acid | covid_severity | Severe | -0.22 | 0.05 | 241 | 241 | 8.62E-06 | 0.000355995 |
| glutamate | read_depth | read_depth | -0.03 | 0.01 | 241 | 241 | 9.44E-06 | 0.000366065 |
| C16.carnitine | covid_severity | Severe | 0.25 | 0.05 | 241 | 241 | 9.40E-06 | 0.000366065 |
| fructose.glucose.galactose. | covid_severity | Severe | 0.13 | 0.03 | 241 | 241 | 1.03E-05 | 0.000385302 |
| trimethyllysine | covid_severity | Severe | 0.17 | 0.04 | 241 | 241 | 1.05E-05 | 0.000385302 |
| inosine | time_from_admit | time_from_admit | 0.00 | 0.00 | 241 | 241 | 1.40E-05 | 0.000496023 |
| nicotinic.acid | time_from_admit | time_from_admit | 0.00 | 0.00 | 241 | 241 | 1.52E-05 | 0.000527136 |
| chenodeoxycholate | covid_severity | Severe | 0.83 | 0.19 | 241 | 241 | 1.64E-05 | 0.000550792 |
| ADMA.SDMA. | covid_severity | Severe | 0.34 | 0.08 | 241 | 241 | 1.97E-05 | 0.000646026 |
| hypoxanthine | covid_severity | Severe | 0.13 | 0.03 | 241 | 241 | 2.24E-05 | 0.000717207 |
| citrulline | read_depth | read_depth | -0.16 | 0.04 | 241 | 241 | 3.33E-05 | 0.001040072 |
| phytosphingosine | covid_severity | Severe | 0.29 | 0.07 | 241 | 241 | 4.77E-05 | 0.001453415 |
| deoxycholic.acid | read_depth | read_depth | 0.08 | 0.02 | 241 | 241 | 5.39E-05 | 0.00160493 |
| X2.hydroxymyristic.acid | covid_severity | Severe | -0.17 | 0.04 | 241 | 241 | 5.67E-05 | 0.00165003 |
| X7.methylguanine | read_depth | read_depth | -0.07 | 0.02 | 241 | 241 | 6.34E-05 | 0.001803744 |
| fructose.glucose.galactose. | read_depth | read_depth | -0.04 | 0.01 | 241 | 241 | 7.56E-05 | 0.002057552 |
| X3.methylxanthine | read_depth | read_depth | -0.07 | 0.02 | 241 | 241 | 7.50E-05 | 0.002057552 |
| X3.methyladipate.pimelate | covid_severity | Severe | -0.81 | 0.20 | 241 | 241 | 8.14E-05 | 0.002171103 |
| trimethyllysine | read_depth | read_depth | -0.05 | 0.01 | 241 | 241 | 8.37E-05 | 0.002185978 |
| ketodeoxycholate | covid_severity | Severe | 0.62 | 0.16 | 241 | 241 | 0.00014028 | 0.003591179 |
| N.acetylspermidine | read_depth | read_depth | -0.04 | 0.01 | 241 | 241 | 0.000144171 | 0.003618403 |
| hypoxanthine | read_depth | read_depth | -0.04 | 0.01 | 241 | 241 | 0.000154438 | 0.003729819 |
| lithocholate | read_depth | read_depth | 0.14 | 0.04 | 241 | 241 | 0.000152802 | 0.003729819 |
| lithocholic.acid | covid_severity | Severe | -0.36 | 0.09 | 241 | 241 | 0.000162452 | 0.003780703 |
| urobilin | read_depth | read_depth | 0.16 | 0.04 | 241 | 241 | 0.000160045 | 0.003780703 |
| malonate | read_depth | read_depth | -0.06 | 0.02 | 241 | 241 | 0.000205 | 0.004648292 |
| N.acetylhistidine | covid_severity | Severe | 0.10 | 0.03 | 241 | 241 | 0.000206994 | 0.004648292 |
| propionate | time_from_admit | time_from_admit | 0.00 | 0.00 | 241 | 241 | 0.000220761 | 0.004871975 |
| ADMA.SDMA. | read_depth | read_depth | -0.10 | 0.03 | 241 | 241 | 0.000224843 | 0.004877953 |
| nicotinate | time_from_admit | time_from_admit | 0.00 | 0.00 | 241 | 241 | 0.00023257 | 0.0049615 |
| pantothenate | read_depth | read_depth | -0.04 | 0.01 | 241 | 241 | 0.00023697 | 0.004972487 |
| deoxyinosine | covid_severity | Severe | -0.10 | 0.03 | 241 | 241 | 0.000241061 | 0.004976737 |
| C16.0.ceramide..d18.1. | read_depth | read_depth | -0.07 | 0.02 | 241 | 241 | 0.000294651 | 0.005893015 |
| dimethyllysine | read_depth | read_depth | -0.06 | 0.02 | 241 | 241 | 0.000293893 | 0.005893015 |
| pyridoxamine | time_from_admit | time_from_admit | -0.01 | 0.00 | 241 | 241 | 0.000308764 | 0.006080272 |
| threosphingosine | covid_severity | Severe | 0.22 | 0.06 | 241 | 241 | 0.000323812 | 0.006279998 |
| C18.0.SM | read_depth | read_depth | -0.06 | 0.02 | 241 | 241 | 0.000393055 | 0.007509101 |
| butyrate...isobutytare. | time_from_admit | time_from_admit | 0.00 | 0.00 | 241 | 241 | 0.000413187 | 0.007777632 |
| N.acetylhistidine | read_depth | read_depth | -0.03 | 0.01 | 241 | 241 | 0.00044346 | 0.008226504 |
| palmitoyl.glycerol | read_depth | read_depth | -0.05 | 0.01 | 241 | 241 | 0.000485578 | 0.008681929 |
| C18.0e.MAG | read_depth | read_depth | -0.05 | 0.02 | 241 | 241 | 0.000495141 | 0.008681929 |
| putrescine | covid_severity | Severe | 0.17 | 0.05 | 241 | 241 | 0.000483709 | 0.008681929 |
| urobilin. | covid_severity | Severe | -0.34 | 0.10 | 241 | 241 | 0.000488661 | 0.008681929 |
| docosahexaenoic.acid | read_depth | read_depth | -0.05 | 0.01 | 241 | 241 | 0.000514905 | 0.008906466 |
| threosphingosine | read_depth | read_depth | -0.08 | 0.02 | 241 | 241 | 0.000529861 | 0.009042959 |
| C18.1.CE | read_depth | read_depth | -0.07 | 0.02 | 241 | 241 | 0.00054283 | 0.0091424 |
| urobilin | covid_severity | Severe | -0.44 | 0.12 | 241 | 241 | 0.000577728 | 0.009603783 |
| lithocholic.acid | read_depth | read_depth | 0.11 | 0.03 | 241 | 241 | 0.000586122 | 0.009618409 |
| N.acetylglutamic.acid | time_from_admit | time_from_admit | 0.00 | 0.00 | 241 | 241 | 0.000620659 | 0.010056251 |
| uracil | read_depth | read_depth | -0.03 | 0.01 | 241 | 241 | 0.000658101 | 0.010529618 |
| thymine | on_abx | TRUE | -0.12 | 0.03 | 241 | 241 | 0.000673312 | 0.010639998 |
| uracil | ethnicity | Hispanic | 0.11 | 0.03 | 241 | 241 | 0.000693054 | 0.010787418 |
| N.acetylglutamate | time_from_admit | time_from_admit | 0.00 | 0.00 | 241 | 241 | 0.000699497 | 0.010787418 |
| X3.methyladipate.pimelate | read_depth | read_depth | 0.24 | 0.07 | 241 | 241 | 0.000754994 | 0.011504669 |
| X7.methylguanine | covid_severity | Severe | 0.17 | 0.05 | 241 | 241 | 0.000812039 | 0.01222835 |
| thymine | time_from_admit | time_from_admit | 0.00 | 0.00 | 241 | 241 | 0.000831015 | 0.012368592 |
| N.acetylglutamic.acid | covid_severity | Severe | -0.14 | 0.04 | 241 | 241 | 0.000858091 | 0.012624793 |
| N.acetylputrescine | read_depth | read_depth | -0.19 | 0.06 | 241 | 241 | 0.00089407 | 0.013004654 |
| alpha.muricholate | covid_severity | Severe | 0.47 | 0.14 | 241 | 241 | 0.000930485 | 0.013233568 |
| nicotinic.acid | covid_severity | Severe | -0.15 | 0.04 | 241 | 241 | 0.00092435 | 0.013233568 |
| X3.methyladipate.pimelate | time_from_admit | time_from_admit | -0.02 | 0.00 | 241 | 241 | 0.000960216 | 0.013506333 |
| chenodeoxycholate.deoxycholate. | read_depth | read_depth | 0.06 | 0.02 | 241 | 241 | 0.000992582 | 0.013809831 |
| erythronic.acid | read_depth | read_depth | -0.05 | 0.01 | 241 | 241 | 0.001095172 | 0.014756004 |
| putrescine | read_depth | read_depth | -0.05 | 0.02 | 241 | 241 | 0.001084304 | 0.014756004 |
| X2.hydroxyphenethylamine | read_depth | read_depth | -0.06 | 0.02 | 241 | 241 | 0.001078365 | 0.014756004 |
| urobilin. | read_depth | read_depth | 0.11 | 0.03 | 241 | 241 | 0.001120246 | 0.014936615 |
| glucurote | read_depth | read_depth | -0.03 | 0.01 | 241 | 241 | 0.001197202 | 0.015798128 |
| imidazole.propionate | time_from_admit | time_from_admit | 0.01 | 0.00 | 241 | 241 | 0.001243777 | 0.01624525 |
| arachidonic.acid | read_depth | read_depth | -0.07 | 0.02 | 241 | 241 | 0.001262994 | 0.016329623 |
| glutamate | ethnicity | Hispanic | 0.07 | 0.02 | 241 | 241 | 0.001626736 | 0.020822219 |
| adrenic.acid | read_depth | read_depth | -0.12 | 0.04 | 241 | 241 | 0.001948452 | 0.024693254 |
| N.acetylglutamic.acid | read_depth | read_depth | 0.04 | 0.01 | 241 | 241 | 0.002040459 | 0.025605757 |
| diacetylspermine | read_depth | read_depth | -0.08 | 0.03 | 241 | 241 | 0.002086323 | 0.025927126 |
| bilirubin | read_depth | read_depth | -0.05 | 0.02 | 241 | 241 | 0.002230297 | 0.027449812 |
| chenodeoxycholate | read_depth | read_depth | -0.18 | 0.06 | 241 | 241 | 0.002289898 | 0.027914943 |
| deoxycholic.acid | covid_severity | Severe | -0.17 | 0.05 | 241 | 241 | 0.002443409 | 0.029505315 |
| C16.0.LPC | read_depth | read_depth | -0.10 | 0.03 | 241 | 241 | 0.002598049 | 0.03107947 |
| methylbutyric.aid...valeric...isovaleric. | ethnicity | Hispanic | 0.09 | 0.03 | 241 | 241 | 0.002827162 | 0.033507111 |
| phenylacetate | ethnicity | Hispanic | 0.10 | 0.03 | 241 | 241 | 0.002939101 | 0.034200445 |
| docosapentaenoate | read_depth | read_depth | -0.05 | 0.02 | 241 | 241 | 0.002938817 | 0.034200445 |
| propionate | covid_severity | Severe | -0.12 | 0.04 | 241 | 241 | 0.002966524 | 0.034208565 |
| butyrate...isobutytare. | covid_severity | Severe | -0.10 | 0.03 | 241 | 241 | 0.003021168 | 0.034222084 |
| lithocholate | covid_severity | Severe | -0.31 | 0.10 | 241 | 241 | 0.003011726 | 0.034222084 |
| X2.hydroxymyristic.acid | time_from_admit | time_from_admit | 0.00 | 0.00 | 241 | 241 | 0.003241801 | 0.036399171 |
| taurine | time_from_admit | time_from_admit | 0.00 | 0.00 | 241 | 241 | 0.00340227 | 0.03786874 |
| N.acetylglutamate | covid_severity | Severe | -0.11 | 0.04 | 241 | 241 | 0.003846444 | 0.042443522 |
| phenylacetate | time_from_admit | time_from_admit | 0.00 | 0.00 | 241 | 241 | 0.004067184 | 0.044495684 |
| phenylacetate | read_depth | read_depth | -0.03 | 0.01 | 241 | 241 | 0.004389526 | 0.047571982 |
| N.acetylputrescine | time_from_admit | time_from_admit | 0.01 | 0.00 | 241 | 241 | 0.004422708 | 0.047571982 |
| nicotinic.acid | tx_remdesivir | 1 | 0.15 | 0.05 | 241 | 241 | 0.004483081 | 0.047819532 |
| hydrocinnamic.acid | read_depth | read_depth | 0.12 | 0.04 | 241 | 241 | 0.004603039 | 0.048693304 |
| chenodeoxycholate | time_from_admit | time_from_admit | 0.01 | 0.00 | 241 | 241 | 0.005141217 | 0.053502095 |
| azelaic.acid | covid_severity | Severe | -0.06 | 0.02 | 241 | 241 | 0.005138647 | 0.053502095 |
| creatine | read_depth | read_depth | -0.12 | 0.04 | 241 | 241 | 0.005252932 | 0.05422381 |
| sebacate | tx_remdesivir | 1 | 0.11 | 0.04 | 241 | 241 | 0.005381804 | 0.055109672 |
| citrulline | time_from_admit | time_from_admit | -0.01 | 0.00 | 241 | 241 | 0.005575493 | 0.056639925 |
| cholate | read_depth | read_depth | -0.19 | 0.07 | 241 | 241 | 0.005819732 | 0.058655568 |
| docosapentaenoic.acid | read_depth | read_depth | -0.06 | 0.02 | 241 | 241 | 0.005970239 | 0.059354859 |
| uracil | on_abx | TRUE | -0.07 | 0.03 | 241 | 241 | 0.006010206 | 0.059354859 |
| inosine | covid_severity | Severe | 0.10 | 0.04 | 241 | 241 | 0.006028228 | 0.059354859 |
| bilirubin | time_from_admit | time_from_admit | 0.00 | 0.00 | 241 | 241 | 0.006748826 | 0.065942732 |
| methylbutyric.aid...valeric...isovaleric. | race | Other | -0.12 | 0.04 | 241 | 241 | 0.00690185 | 0.066780861 |
| citrulline | race | Mixed | 0.56 | 0.20 | 241 | 241 | 0.006938949 | 0.066780861 |
| N.acetylhistidine | ethnicity | Hispanic | 0.08 | 0.03 | 241 | 241 | 0.007012465 | 0.066984738 |
| phytosphingosine | read_depth | read_depth | -0.06 | 0.02 | 241 | 241 | 0.007402214 | 0.070183951 |
| xanthine | covid_severity | Severe | 0.10 | 0.04 | 241 | 241 | 0.008634912 | 0.081269758 |
| X3.methylxanthine | covid_severity | Severe | 0.13 | 0.05 | 241 | 241 | 0.008781837 | 0.082049277 |
| cytosine | covid_severity | Severe | -0.20 | 0.08 | 241 | 241 | 0.009102144 | 0.084425683 |
| cholesterol | read_depth | read_depth | -0.06 | 0.02 | 241 | 241 | 0.009435542 | 0.086888447 |
| pseudouridine | ethnicity | Hispanic | 0.06 | 0.02 | 241 | 241 | 0.009826644 | 0.089843606 |
| C16.carnitine | read_depth | read_depth | -0.05 | 0.02 | 241 | 241 | 0.010585145 | 0.096092096 |
| hypoxanthine | ethnicity | Hispanic | 0.08 | 0.03 | 241 | 241 | 0.011708437 | 0.105540844 |
| sebacate | covid_severity | Severe | -0.09 | 0.03 | 241 | 241 | 0.012193619 | 0.109145682 |
| cholesterol | race | Asian | 0.35 | 0.14 | 241 | 241 | 0.012816658 | 0.113925845 |
| phenylacetate | race | Other | -0.11 | 0.04 | 241 | 241 | 0.013178704 | 0.116336142 |
| butyrate...isobutytare. | race | Other | -0.13 | 0.05 | 241 | 241 | 0.014286727 | 0.122744707 |
| N.acetylglutamate | read_depth | read_depth | 0.03 | 0.01 | 241 | 241 | 0.014288251 | 0.122744707 |
| X2.hydroxyphenethylamine | time_from_admit | time_from_admit | 0.00 | 0.00 | 241 | 241 | 0.014191965 | 0.122744707 |
| ADMA | read_depth | read_depth | -0.09 | 0.04 | 241 | 241 | 0.014123795 | 0.122744707 |
| pyridoxamine | age | age | -0.07 | 0.03 | 241 | 241 | 0.014941382 | 0.127499792 |
| pantothenate | ethnicity | Hispanic | 0.08 | 0.03 | 241 | 241 | 0.015604519 | 0.13227672 |
| chenodeoxycholate.deoxycholate. | covid_severity | Severe | -0.11 | 0.04 | 241 | 241 | 0.015847332 | 0.133451221 |
| cholate | time_from_admit | time_from_admit | 0.01 | 0.00 | 241 | 241 | 0.016180878 | 0.135369435 |
| butyrate...isobutytare. | ethnicity | Hispanic | 0.09 | 0.04 | 241 | 241 | 0.016962439 | 0.140986502 |
| nicotinate | ethnicity | Hispanic | 0.05 | 0.02 | 241 | 241 | 0.017291782 | 0.142796649 |
| citrulline | covid_severity | Severe | 0.23 | 0.10 | 241 | 241 | 0.0174318 | 0.143030156 |
| deoxyinosine | tx_remdesivir | 1 | 0.07 | 0.03 | 241 | 241 | 0.018183261 | 0.147485032 |
| trimethyllysine | ethnicity | Hispanic | 0.10 | 0.04 | 241 | 241 | 0.018205184 | 0.147485032 |
| pseudouridine | time_from_admit | time_from_admit | 0.00 | 0.00 | 241 | 241 | 0.01834276 | 0.147664985 |
| stearoyl.ethanolamide | covid_severity | Severe | 0.12 | 0.05 | 241 | 241 | 0.018515443 | 0.148123541 |
| eicosatrienoic.acid | read_depth | read_depth | -0.08 | 0.03 | 241 | 241 | 0.018875582 | 0.150066742 |
| alpha.muricholate | read_depth | read_depth | -0.12 | 0.05 | 241 | 241 | 0.019746625 | 0.156022713 |
| undecanedionate | read_depth | read_depth | 0.08 | 0.03 | 241 | 241 | 0.020261516 | 0.158894727 |
| eicosatrienoic.acid | on_abx | TRUE | 0.23 | 0.10 | 241 | 241 | 0.020358387 | 0.158894727 |
| linoleoyl.ethanolamide | race | Asian | 0.29 | 0.12 | 241 | 241 | 0.021418726 | 0.164167481 |
| pseudouridine | on_abx | TRUE | -0.05 | 0.02 | 241 | 241 | 0.021333498 | 0.164167481 |
| urobilin. | time_from_admit | time_from_admit | 0.00 | 0.00 | 241 | 241 | 0.021348001 | 0.164167481 |
| N.acetylglutamic.acid | tx_remdesivir | 1 | 0.11 | 0.05 | 241 | 241 | 0.021731141 | 0.1655706 |
| fructose.glucose.galactose. | ethnicity | Hispanic | 0.07 | 0.03 | 241 | 241 | 0.022479526 | 0.170259131 |
| pseudouridine | read_depth | read_depth | -0.02 | 0.01 | 241 | 241 | 0.022631406 | 0.170401177 |
| docosahexaenoic.acid | ethnicity | Hispanic | 0.10 | 0.04 | 241 | 241 | 0.023573815 | 0.17543304 |
| phenylacetate | covid_severity | Severe | 0.07 | 0.03 | 241 | 241 | 0.023463317 | 0.17543304 |
| ketodeoxycholate | time_from_admit | time_from_admit | 0.01 | 0.00 | 241 | 241 | 0.023884267 | 0.176715965 |
| adrenic.acid | time_from_admit | time_from_admit | 0.01 | 0.00 | 241 | 241 | 0.024328867 | 0.177210196 |
| propionate | read_depth | read_depth | -0.03 | 0.01 | 241 | 241 | 0.024366402 | 0.177210196 |
| cytosine | ethnicity | Hispanic | 0.20 | 0.09 | 241 | 241 | 0.024147941 | 0.177210196 |
| C18.0e.MAG | time_from_admit | time_from_admit | 0.00 | 0.00 | 241 | 241 | 0.024953079 | 0.180451644 |
| ketodeoxycholate | age | age | 0.18 | 0.08 | 241 | 241 | 0.025103284 | 0.180517998 |
| glucurote | ethnicity | Hispanic | 0.06 | 0.03 | 241 | 241 | 0.02535437 | 0.181304992 |
| azelate | time_from_admit | time_from_admit | 0.00 | 0.00 | 241 | 241 | 0.026171745 | 0.186110184 |
| azelate | read_depth | read_depth | 0.03 | 0.02 | 241 | 241 | 0.026599697 | 0.187074794 |
| caproic.acid | read_depth | read_depth | 0.04 | 0.02 | 241 | 241 | 0.026535835 | 0.187074794 |
| deoxycholic.acid | ethnicity | Hispanic | -0.13 | 0.06 | 241 | 241 | 0.027002475 | 0.187843302 |
| imidazole.propionate | read_depth | read_depth | -0.07 | 0.03 | 241 | 241 | 0.026933653 | 0.187843302 |
| palmitoyl.glycerol | ethnicity | Hispanic | 0.11 | 0.05 | 241 | 241 | 0.02798757 | 0.193643729 |
| C18.0e.MAG | ethnicity | Hispanic | 0.11 | 0.05 | 241 | 241 | 0.028688779 | 0.197428159 |
| pyridoxamine | read_depth | read_depth | 0.05 | 0.02 | 241 | 241 | 0.028891782 | 0.197761933 |
| imidazole.propionate | on_abx | TRUE | 0.19 | 0.09 | 241 | 241 | 0.030439837 | 0.207249953 |
| urobilin | time_from_admit | time_from_admit | -0.01 | 0.00 | 241 | 241 | 0.030964308 | 0.209290399 |
| N.acetylglutamate | tx_remdesivir | 1 | 0.09 | 0.04 | 241 | 241 | 0.031066544 | 0.209290399 |
| glutamate | covid_severity | Severe | 0.04 | 0.02 | 241 | 241 | 0.031418353 | 0.210552311 |
| N.oleoylethanolamine | race | Asian | 0.44 | 0.20 | 241 | 241 | 0.031618631 | 0.210790876 |
| nicotinate | read_depth | read_depth | -0.02 | 0.01 | 241 | 241 | 0.032675318 | 0.216706772 |
| propionate | male | yes | -0.07 | 0.03 | 241 | 241 | 0.033058138 | 0.218115551 |
| threosphingosine | ethnicity | Hispanic | 0.14 | 0.07 | 241 | 241 | 0.035235592 | 0.231290042 |
| threosphingosine | time_from_admit | time_from_admit | 0.00 | 0.00 | 241 | 241 | 0.035545903 | 0.232136511 |
| docosapentaenoic.acid | time_from_admit | time_from_admit | 0.00 | 0.00 | 241 | 241 | 0.036463818 | 0.23427333 |
| malonate | ethnicity | Hispanic | 0.11 | 0.05 | 241 | 241 | 0.03656699 | 0.23427333 |
| uracil | time_from_admit | time_from_admit | 0.00 | 0.00 | 241 | 241 | 0.036439648 | 0.23427333 |
| cytosine | race | Other | -0.26 | 0.12 | 241 | 241 | 0.036605208 | 0.23427333 |
| hydrocinnamic.acid | covid_severity | Severe | -0.26 | 0.12 | 241 | 241 | 0.038445241 | 0.244825415 |
| undecanedionate | time_from_admit | time_from_admit | 0.00 | 0.00 | 241 | 241 | 0.039082056 | 0.247648675 |
| stearoyl.ethanolamide | race | Asian | 0.22 | 0.11 | 241 | 241 | 0.039685653 | 0.249257849 |
| erythronic.acid | ethnicity | Hispanic | 0.09 | 0.04 | 241 | 241 | 0.03972547 | 0.249257849 |
| Results with FDR-corrected p-value <0.25 shown. | | | | | | | | |

**Supplementary Figure 1. Volcano plot for multivariable linear modeling results (MetaCyc pathways)**

**
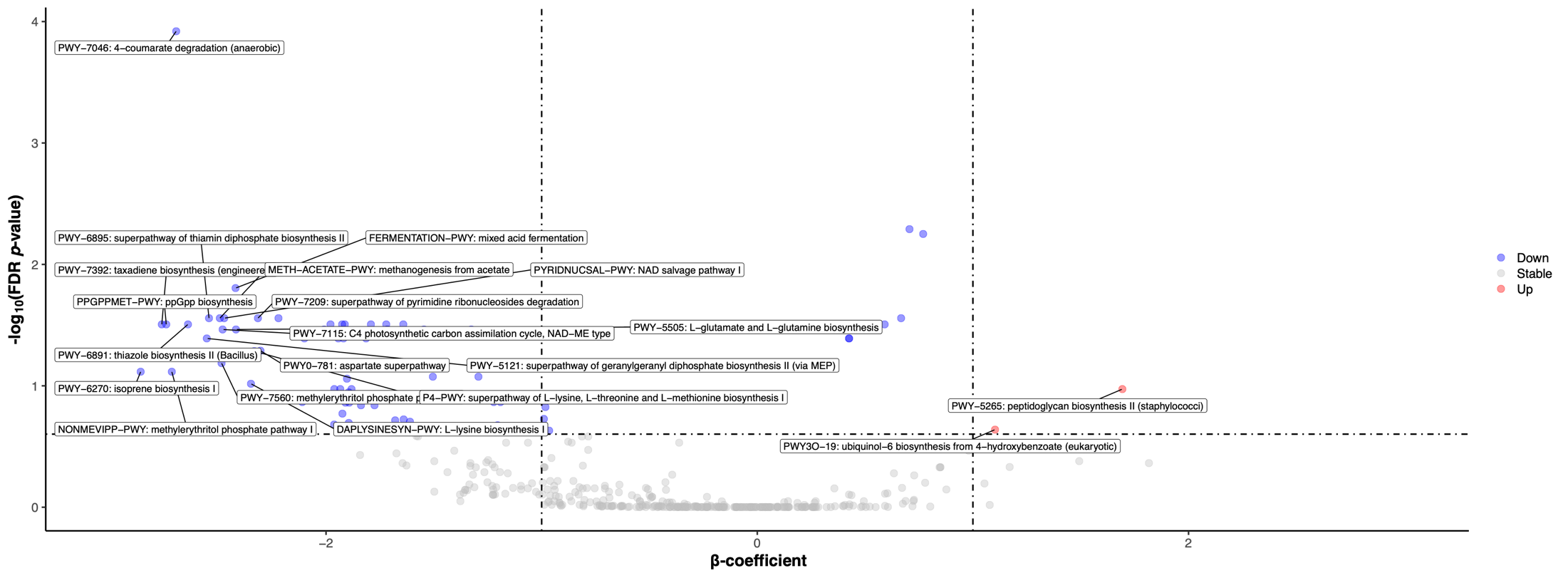
**

**Supplementary Figure 2. Entropy heatmap for clinical covariates**

**
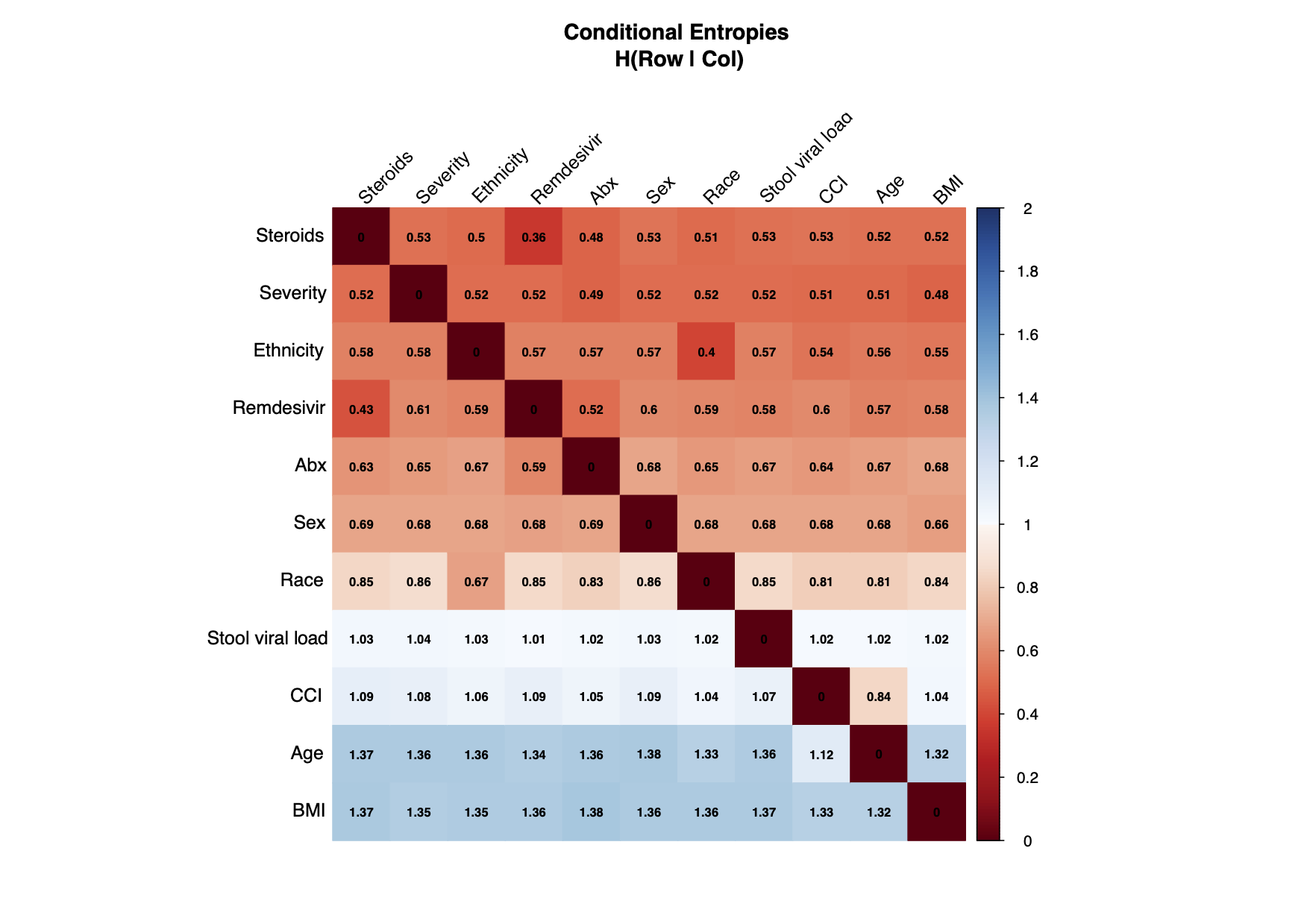
**

**Supplementary Figure 3. Node map**


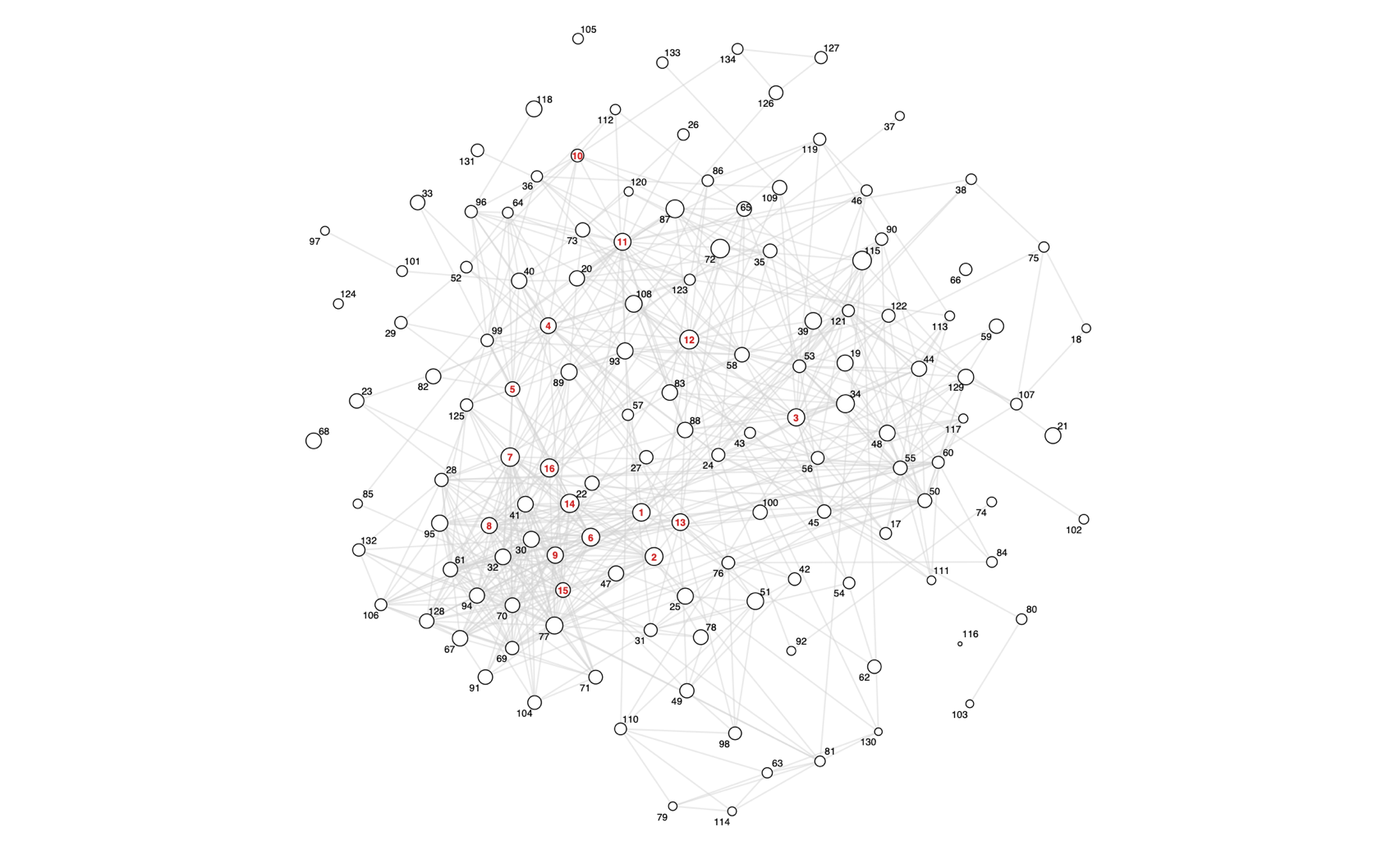


| **Number** | **Species** | **Hub Status** |
| --- | --- | --- |
| 1 | *Blautia wexlerae* | Hub |
| 2 | *Eubacterium hallii* | Hub |
| 3 | *Gordonibacter pamelaeae* | Hub |
| 4 | *Odoribacter splanchnicus* | Hub |
| 5 | *Alistipes shahii* | Hub |
| 6 | *Anaerostipes hadrus* | Hub |
| 7 | *Faecalibacterium prausnitzii* | Hub |
| 8 | *Dorea formicigenerans* | Hub |
| 9 | *Eubacterium rectale* | Hub |
| 10 | *Bacteroides massiliensis* | Hub |
| 11 | *Alistipes putredinis* | Hub |
| 12 | *Bacteroides uniformis* | Hub |
| 13 | *Streptococcus salivarius* | Hub |
| 14 | *Collinsella aerofaciens* | Hub |
| 15 | *Roseburia inulinivorans* | Hub |
| 16 | *Blautia obeum* | Hub |
| 17 | *Clostridium spiroforme* |  |
| 18 | *Enterococcus faecalis* |  |
| 19 | *Clostridium leptum* |  |
| 20 | *Parabacteroides merdae* |  |
| 21 | *Streptococcus thermophilus* |  |
| 22 | *Collinsella stercoris* |  |
| 23 | *Akkermansia muciniphila* |  |
| 24 | *Anaerotruncus colihominis* |  |
| 25 | *Bifidobacterium longum* |  |
| 26 | *Clostridium citroniae* |  |
| 27 | *Intestinimonas butyriciproducens* |  |
| 28 | *Oscillibacter* sp. CAG 241 |  |
| 29 | *Phascolarctobacterium faecium* |  |
| 30 | *Ruminococcus torques* |  |
| 31 | *Streptococcus vestibularis* |  |
| 32 | *Eubacterium eligens* |  |
| 33 | *Bacteroides dorei* |  |
| 34 | *Eggerthella lenta* |  |
| 35 | *Lawsonibacter asaccharolyticus* |  |
| 36 | *Holdemania filiformis* |  |
| 37 | *Dielma fastidiosa* |  |
| 38 | *Enterococcus faecium* |  |
| 39 | *Flavonifractor plautii* |  |
| 40 | *Methanobrevibacter smithii* |  |
| 41 | *Roseburia hominis* |  |
| 42 | *Actinomyces oris* |  |
| 43 | *Anaerofustis stercorihominis* |  |
| 44 | *Blautia hydrogenotrophica* |  |
| 45 | *Dorea sp.* CAG 317 |  |
| 46 | *Harryflintia acetispora* |  |
| 47 | *Ruminococcus bromii* |  |
| 48 | *Ruminococcus gnavus* |  |
| 49 | *Actinomyces odontolyticus* |  |
| 50 | *Blautia sp.* CAG 257 |  |
| 51 | *Streptococcus parasanguinis* |  |
| 52 | *Parabacteroides goldsteinii* |  |
| 53 | *Eisenbergiella massiliensis* |  |
| 54 | *Clostridium clostridioforme* |  |
| 55 | *Sellimonas intestinalis* |  |
| 56 | *Monoglobus pectinilyticus* |  |
| 57 | *Streptococcus anginosus group* |  |
| 58 | *Eisenbergiella tayi* |  |
| 59 | *Erysipelatoclostridium ramosum* |  |
| 60 | *Blautia producta* |  |
| 61 | *Gemmiger formicilis* |  |
| 62 | *Bacteroides fragilis* |  |
| 63 | *Lactobacillus paragasseri* |  |
| 64 | *Bacteroides salyersiae* |  |
| 65 | *Bacteroides stercoris* |  |
| 66 | *Hungatella hathewayi* |  |
| 67 | *Agathobaculum butyriciproducens* |  |
| 68 | *Escherichia coli* |  |
| 69 | *Oscillibacter sp.* 57 20 |  |
| 70 | *Coprococcus catus* |  |
| 71 | *Roseburia intestinalis* |  |
| 72 | *Bacteroides vulgatus* |  |
| 73 | *Bacteroides xylanisolvens* |  |
| 74 | *Clostridium asparagiforme* |  |
| 75 | *Lactobacillus rhamnosus* |  |
| 76 | *Streptococcus gordonii* |  |
| 77 | *Fusicatenibacter saccharivorans* |  |
| 78 | *Firmicutes bacterium* CAG 83 |  |
| 79 | *Lactobacillus gasseri* |  |
| 80 | *Bacteroides faecis* |  |
| 81 | *Lactobacillus fermentum* |  |
| 82 | *Eubacterium siraeum* |  |
| 83 | *Adlercreutzia equolifaciens* |  |
| 84 | *Anaerotignum lactatifermentans* |  |
| 85 | *Bacteroides nordii* |  |
| 86 | *Clostridium lavalense* |  |
| 87 | *Parabacteroides distasonis* |  |
| 88 | *Asaccharobacter celatus* |  |
| 89 | *Bacteroides thetaiotaomicron* |  |
| 90 | *Clostridium symbiosum* |  |
| 91 | *Bifidobacterium adolescentis* |  |
| 92 | *Ruminococcaceae bacterium* D16 |  |
| 93 | *Bacteroides ovatus* |  |
| 94 | *Coprococcus comes* |  |
| 95 | *Dorea longicatena* |  |
| 96 | *Butyricimonas virosa* |  |
| 97 | *Eubacterium callanderi* |  |
| 98 | *Rothia mucilaginosa* |  |
| 99 | *Alistipes indistinctus* |  |
| 100 | *Bilophila wadsworthia* |  |
| 101 | *Eubacterium limosum* |  |
| 102 | *Klebsiella pneumoniae* |  |
| 103 | *Bacteroides faecis* CAG 32 |  |
| 104 | *Roseburia faecis* |  |
| 105 | *Paraprevotella xylaniphila* |  |
| 106 | *Prevotella copri* |  |
| 107 | *Blautia coccoides* |  |
| 108 | *Alistipes finegoldii* |  |
| 109 | *Clostridium bolteae* |  |
| 110 | *Veillonella parvula* |  |
| 111 | *Clostridium hylemonae* |  |
| 112 | *Bacteroides finegoldii* |  |
| 113 | *Clostridium methylpentosum* |  |
| 114 | *Bifidobacterium breve* |  |
| 115 | *Ruthenibacterium lactatiformans* |  |
| 116 | *Faecalicatena orotica* |  |
| 117 | *Anaerostipes caccae* |  |
| 118 | *Bacteroides caccae* |  |
| 119 | *Bacteroides cellulosilyticus* |  |
| 120 | *Catabacter hongkongensis* |  |
| 121 | *Clostridium scindens* |  |
| 122 | *Firmicutes bacterium* CAG 145 |  |
| 123 | *Butyricimonas synergistica* |  |
| 124 | *Christensenella minuta* |  |
| 125 | *Clostridium* sp. CAG 58 |  |
| 126 | *Parasutterella excrementihominis* |  |
| 127 | *Proteobacteria bacterium* CAG 139 |  |
| 128 | *Eubacterium ramulus* |  |
| 129 | *Clostridium innocuum* |  |
| 130 | *Candida albicans* |  |
| 131 | *Barnesiella intestinihominis* |  |
| 132 | *Holdemanella biformis* |  |
| 133 | *Clostridium bolteae* CAG 59 |  |
| 134 | *Turicimonas muris* |  |
| NODE IDENTITIES MAPPED ON MODERATE NETWORK (NOTE: NODE POSITION BETWEEN MODERATE AND SEVERE NETWORKS ARE LOCKED) | | |

**Supplementary Figure 4. PCoA by batch**

**
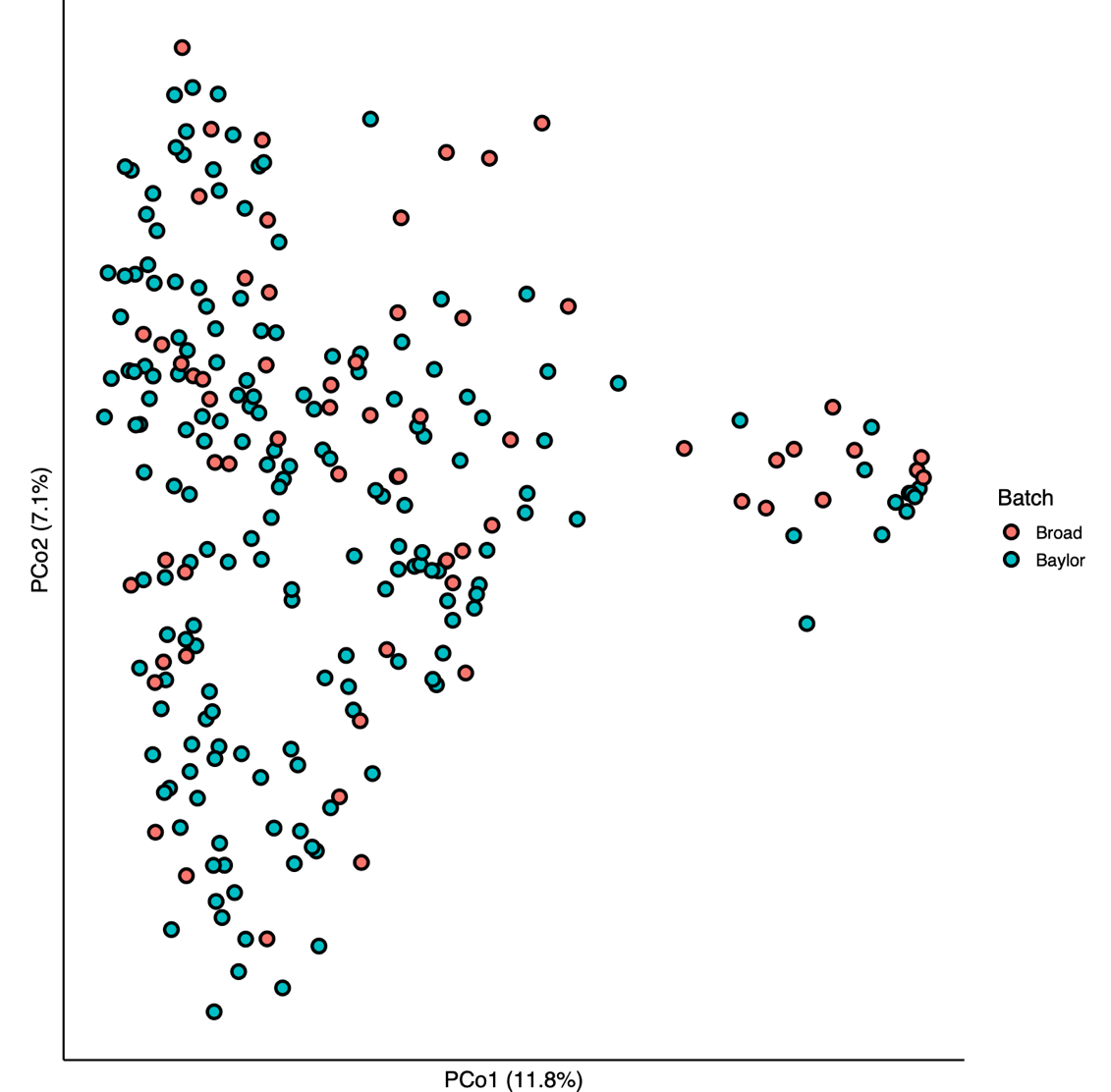
**
